# GM604 regulates developmental neurogenesis pathways and the expression of genes associated with amyotrophic lateral sclerosis

**DOI:** 10.1186/s40035-018-0135-7

**Published:** 2018-12-03

**Authors:** William R. Swindell, Krzysztof Bojanowski, Mark S. Kindy, Raymond M. W. Chau, Dorothy Ko

**Affiliations:** 1 Heritage College of Osteopathic Medicine, Ohio University, Athens, OH USA; 2 Sunny BioDiscovery, Inc, Santa Paula, CA USA; 3 Department of Pharmaceutical Sciences, College of Pharmacy, University of South Florida, Tampa, FL USA; 4 James A. Haley VAMC, Tampa, FL USA; 5 Genervon Biopharmaceuticals LLC, Pasadena, CA USA

**Keywords:** Alzheimer’s disease, Amyotrophic lateral sclerosis, Extracellular matrix, Hedgehog, Huntington’s disease, Neurodegenerative disease, Notch, Parkinson’s disease, RNA-seq, Transcriptome

## Abstract

**Background:**

Amyotrophic lateral sclerosis (ALS) is currently an incurable disease without highly effective pharmacological treatments. The peptide drug GM604 (GM6 or Alirinetide) was developed as a candidate ALS therapy, which has demonstrated safety and good drug-like properties with a favorable pharmacokinetic profile. GM6 is hypothesized to bolster neuron survival through the multi-target regulation of developmental pathways, but mechanisms of action are not fully understood.

**Methods:**

This study used RNA-seq to evaluate transcriptome responses in SH-SY5Y neuroblastoma cells following GM6 treatment (6, 24 and 48 h).

**Results:**

We identified 2867 protein-coding genes with expression significantly altered by GM6 (FDR < 0.10). Early (6 h) responses included up-regulation of Notch and hedgehog signaling components, with increased expression of developmental genes mediating neurogenesis and axon growth. Prolonged GM6 treatment (24 and 48 h) altered the expression of genes contributing to cell adhesion and the extracellular matrix. GM6 further down-regulated the expression of genes associated with mitochondria, inflammatory responses, mRNA processing and chromatin organization. GM6-increased genes were located near GC-rich motifs interacting with C2H2 zinc finger transcription factors, whereas GM6-decreased genes were located near AT-rich motifs associated with helix-turn-helix homeodomain factors. Such motifs interacted with a diverse network of transcription factors encoded by GM6-regulated genes (*STAT3*, *HOXD11*, *HES7*, *GLI1*). We identified 77 ALS-associated genes with expression significantly altered by GM6 treatment (FDR < 0.10), which were known to function in neurogenesis, axon guidance and the intrinsic apoptosis pathway.

**Conclusions:**

Our findings support the hypothesis that GM6 acts through developmental-stage pathways to influence neuron survival. Gene expression responses were consistent with neurotrophic effects, ECM modulation, and activation of the Notch and hedgehog neurodevelopmental pathways. This multifaceted mechanism of action is unique among existing ALS drug candidates and may be applicable to multiple neurodegenerative diseases.

**Electronic supplementary material:**

The online version of this article (10.1186/s40035-018-0135-7) contains supplementary material, which is available to authorized users.

## Background

Amyotrophic lateral sclerosis (ALS or “Lou Gehrig” disease) is currently an incurable heterogeneous disease of unknown etiology characterized by motor neuron death leading to muscle paralysis [1]. The disease most commonly occurs between the ages of 50 and 70 and is twice as common in men compared to women [1]. It is a unique disorder with deficits impacting both lower and upper motor neurons, although either lower or upper motor neuron dysfunction may be dominant for any one patient [2]. The disease is ultimately fatal and death typically occurs from respiratory failure in 1 to 6 years [3]. At present, there is strong urgency among ALS researchers and the patient community to develop effective disease-modifying treatments. To this point, however, clinical ALS management has emphasized supportive measures (e.g., muscle relaxants) and working closely with patients to preserve physiological function (e.g., speech therapy) [4]. A wide range of novel treatments based upon differing concepts have been advanced in pre-clinical research and clinical trials, although promising treatments have often failed to demonstrate efficacy in late-phase clinical studies [5]. In the United States, only three drugs are approved to treat ALS, i.e., riluzole (Rilutek/Teglutik), edaravone (Radicava/Radicut) and dextromethorphan HBr with quinidine sulfate (Nuedexta). Nuedexta is narrowly indicated for treatment of pseudobulbar affect and bulbar symptoms [6], whereas riluzole and edaravone are expected to improve disease course slightly through different mechanisms, i.e., inhibition of glutamate signaling and oxidative stress, respectively [7, 8]. Unfortunately, no currently approved treatment is expected to substantially alter disease course and existing therapies appear to provide only marginal symptomatic benefits [7, 8].

GM604 (GM6 or Alirinetide) is a cationic linear peptide drug (799 Da) that has been developed by Genervon Biopharmaceuticals (Pasadena, CA) [9, 10]. The peptide consists of 6 amino acids (H-Phe-Ser-Arg-Tyr-Ala-Arg-OH) representing a subunit of an endogenous 33 amino acid developmental-stage neurotrophic factor [11] discovered in rat muscle and originally designated as motoneuronotrophic factor 1 (MNTF1) [10, 12–14]. An orthologous human protein was subsequently cloned from a retinoblastoma cDNA library and analyzed to determine its amino acid and cDNA sequences [10, 15]. The peptide drug GM6 is a 6 amino acid analog of the MNTF1 active site that is able to cross the blood-brain barrier [9] and appears to retain functional activity of the full-length protein [16]. GM6 was shown to have good drug-like properties based upon pharmacokinetic profiling, with an estimated volume of distribution of 7.8 L/kg and minimum effective concentration of 1.6 mg/kg in humans [10, 17]. Although the half-life of GM6 in human blood appears short (15 min), the drug partitions readily into tissues where the half-life was estimated to be 5.8 h, with an expected brain to plasma concentration ratio of 1.65 [10]. Cellular uptake of GM6 has been directly demonstrated using quantitative imaging in induced pluripotent stem cell-derived GABAergic neurons, and liver microsome assays further indicated that its clearance rate was unaffected by Riluzole [10]. Recently, a multi-center phase IIA clinical trial was reported in which outcomes were compared between 8 ALS patients receiving GM6 and 4 patients receiving placebo for a 2 week period (ClinicalTrials.gov identifier: NCT01854294) [18]. Although findings from this study do not yet demonstrate efficacy, this trial has demonstrated safety in ALS patients with encouraging trends related to ALS Functional Rating Scale (ALSFRS), forced vital capacity, and ALS biomarkers (e.g., TDP-43, tau protein and SOD1) [18].

GM6 was developed as a multi-target drug believed to trigger developmental-stage signaling pathways, which may be largely dormant in the adult nervous system, but can nonetheless function during the course of development to enhance the survival and growth of neurons [10, 12–14]. The regenerative capacities of the developing nervous system are well known, although the reason why neurons lose this regenerative capacity after development is not fully understood [19]. It has been proposed that drugs designed to restore the developmental gene expression program can provide an avenue for developing neurodegenerative disease treatments [19]. Consistent with this idea, GM6 is modeled upon a neurotrophic factor protein able to promote neurite outgrowth, as demonstrated by trophic effects in transected rat peripheral nerves and neuroprotection against toxic agents in zebrafish [9]. GM6 was also reported to protect against ischemia in a reperfusion injury mouse model [9]. This activity spectrum appears consistent with a classical neurotrophic factor [11], and accordingly, GM6 is expected to have a complex mechanism of action potentially involving stimulation of multiple receptors, signaling cascades and downstream gene expression responses [20]. At present, however, exact mechanisms of action (MOA) for GM6 have not been determined. To develop hypotheses regarding the MOA of investigational drugs such as GM6, transcriptome profiling combined with bioinformatic analysis offers an increasingly powerful approach that can provide a global and objective view of a drug’s cellular effects [21–23]. This approach is especially well-suited to multi-target drugs not developed to specifically interact with one receptor, which may instead interact with multiple receptors with involvement of multiple signaling pathways [20]. To understand the MOA for such drug products, transcriptome profiling provides a valuable tool that can then be used to guide hypothesis-driven studies into one or more drug mechanisms [21–23].

This study used whole transcriptome shotgun sequencing (RNA-seq) to evaluate effects of GM6 on gene expression in the SH-SY5Y neuroblastoma cell line. The SH-SY5Y cell line was here chosen as a flexible model system that has frequently been used in mechanistic studies of ALS and other neurodegenerative diseases [24–29]. We used RNA-seq as an unbiased methodology to fully elucidate the set of genes exhibiting transcriptional responses to GM6 stimulation, with the purpose of identifying effector genes and their controlling upstream signaling components (i.e., extracellular receptors, signaling cascades, transcription factors (TFs) and DNA response elements). Furthermore, given that GM6 is being actively investigated as an ALS therapeutic, we evaluated its effects on the expression of ALS-associated genes. Our findings allow us to propose mechanisms of action to explain neurotrophic effects of GM6 and to hypothesize ways in which these mechanisms may help to preserve motor neuron function in ALS patients.

## Materials & methods

### SH-SY5Y neuroblastoma cells treated with GM6 for 6, 24 and 48 h

SH-SY5Y neuroblastoma cells were treated with GM6 or water (CTL) with independent replicates at 6 h (*n* = 5 GM6, *n* = 3 CTL), 24 h (*n* = 5 GM6, *n* = 5 CTL) and 48 h (*n* = 5 GM6, *n* = 5 CTL). Experiments were performed at Sunny BioDiscovery laboratories (Santa Paula, CA). GM6 hexapeptide was dissolved in cell culture medium and tested at a final concentration of 1 mg/ml (1 mM) for incubation times of 6, 24 or 48 h. Subconfluent SH-SY5Y cells (Sigma, St. Louis, MO) were incubated with GM6 in supplemented Eagle’s Minimum Essential/F12 (1:1) Medium. Experiments were terminated after microscopic observation of cells through Nikon (Tokyo, Japan) Eclipse TS100 inverted microscope. RNA extractions were performed using the NucleoSpin RNA II kit (Machery-Nagel; Bethlehem, PA) with the DNA digestion step and robotic Qiacube (Qiagen, Valencia, CA) workstation. Purified total RNA was assessed at 260 nm and 280 nm with a NanoDrop Lite (Thermo Fisher Scientific, Waltham, MA) spectrophotometer.

### cDNA sequencing and data processing

The SH-SY5Y experiments generated 28 RNA samples submitted for complementary DNA sequencing (University of Michigan sequencing core facility). Sequencing was performed with polyA-selected libraries using a 50 cycle single end Illumina HiSeq 4000 platform. Raw fastq files containing 50 base pair single-end non-stranded reads with phred 33 quality score encoding were provided by the core facility in January and February of 2017. Reads were combined across sequencing runs for each of 28 sequencing samples. Cutadapt was used to remove Illumina adaptor sequence (AGATCGGAAGAGC) with maximum error rate (−e) of 5% and minimum read length (−m) of 20 base pairs [30]. To remove sequences mapping to rRNA, an initial tophat2 run [31] was carried out using an edited gtf file specifying only rRNA sequence coordinates for the UCSC GRCh38/hg38 genome sequence, with 1 transcriptome alignment reported per read (−g 1 --transcriptome-only) and disabling of coverage-based junction search (−-no-coverage-search). Further analyses were then performed using only the unmapped reads from this preliminary tophat2 run. Cutadapt was used to trim reads from the 3′ end using a quality score threshold of 30 (−q) and minimum read length of 20. Reads were then filtered using the Fastx toolkit function fastq_quality_filter to retain only those reads with quality scores above 30 for at least 50% of base pairs (settings: -q 30 -p 50) [32]. Read tabulation and quality analyses before and after filtering were performed using FastQC [33] and the Fastx toolkit function fastx_quality_stats [32].

After read filtering was complete, tophat2 was used to map remaining reads to the UCSC GRCh38/hg38 transcriptome (−-transcriptome-only) with disabling of read multi-mapping (−g 1) and coverage-based junction search (−-no-coverage-search) [31]. Alignment files generated from the tophat2 run for each sample were indexed and sorted using samtools [34]. Read counts for each GRCh38/hg38 human gene were tabulated using htseq-count [35], with reads assigned to a gene only when the alignment quality was greater than 10 (−a 10) and when the read completely and unambiguously overlapped a gene’s sequence (−m intersection-strict). Fragments per kilobase of exon per million reads mapped (FPKM) values and 95% confidence intervals were calculated using Cufflinks with default settings [36]. RNA-SeQC was used to calculate the proportion of reads mapped to ribosomal genes, introns, exons and intergenic sequences [37].

Following removal of reads mapping to rRNA and reads with low quality scores, we obtained an average of 54.8 million reads per sample (Additional File 1A). Of these, 97.8% on average mapped to the UCSC GRCh38/hg38 genome sequence (Additional file 1 B), with 89.1% assigned to intragenic sequences (Additional file 1 C) and 82.3% assigned to exons (Additional file 1 D). As expected from our read filtering protocol, only 0.13% of reads on average aligned to ribosomal RNA (Additional file 1 E). An average of 14,299 protein-coding genes were detected among the 28 samples (Additional file 1 F). Since SH-SY5Y cells are derived from a female donor [25], we expected that few reads would map to chromosome Y. Consistent with this, the average FPKM of protein-coding genes on chromosome Y (0.10) was substantially less than that of protein-coding genes on other chromosomes (average FPKM ≥11.7) (Additional file 2 A). Protein-coding genes on chromosome Y had detectable expression in only 13% of samples on average (compared to ≥57% for other chromosomes; Additional file 2 B), with residual mapping to Y likely explained by low complexity DNA sequence or paralogous regions on sex chromosomes [38]. The 28 samples were clustered based on the expression of protein-coding genes and plotted with respect to the first two principal component axes (Additional file 3). One sample was identified as an outlier in both cluster and principal component analyses (i.e., CTL-48 h-1; Additional file 3). The same sample also differed notably from others with a lower percentage of reads mapped (89.3%) and fewer protein-coding genes with detectable expression (13747) (Additional file 1 B and F). The sample CTL-48 h-1 was therefore excluded and subsequent analyses were based upon the 27 remaining samples.

### Differential expression analyses

Differential expression analyses were performed to compare expression of protein-coding genes between GM6 and CTL cells at the 6, 24 and 48 h time points, respectively. An additional differential expression analyses was carried out to compare GM6 and CTL treatments with samples combined across time points (6–48 h). Differential expression analyses were performed using only protein-coding genes with detectable expression in at least 25% of the samples included in a given GM6 vs. CTL comparison. For a given sample, a gene was considered to have detectable expression if at least 1 read mapped to its sequence and if the FPKM 95% confidence interval lower bound was greater than 0. Applying these criteria, differential expression analyses were performed for 13,736, 13,887 and 13,970 protein-coding genes at the 6, 24 and 48 h time points, respectively. Likewise, differential expression analyses were performed for 14,813 protein-coding genes in the combined analysis (6–48 h).

The negative binomial model and likelihood ratio test approach implemented in edgeR was used to evaluate differential expression for each comparison (functions: glmFit and glmLRT) [39, 40]. Read counts were normalized using the weighted trimmed mean of M-values method [41] with dispersions estimated using the Cox-Reid (CR)-adjusted likelihood approach [40]. For comparisons at a single time point (6, 24 or 48 h), a design matrix was constructed based upon a single treatment variable indicating whether samples belonged to the GM6 or CTL treatment (full model). Likelihood ratio tests were then performed by dropping the treatment variable (reduced model) and comparing likelihood between the two models (full vs. reduced). For the combined analysis (6–48 h), the design matrix was constructed with one treatment variable (GM6 vs. CTL) and a second variable indicating treatment time (6, 24 and 48 h) (full model). Likelihood ratio tests were again performed by dropping the treatment variable (reduced model) and comparing likelihood between the two models (full vs. reduced). To control the false discovery rate, raw *p*-values generated from likelihood ratio tests were adjusted using the Benjamini-Hochberg method [42].

### Gene ontology and pathway analyses

To characterize functional themes among differentially expressed genes, we assessed identified genes for enrichment of annotations with respect to multiple database sources, including Gene Ontology (GO) [43], Kyoto Encyclopedia of Genes and Genomes (KEGG) [44], Reactome [45], and Disease Ontology [46]. Enrichment of GO biological process, GO cell component and KEGG terms was evaluated using the conditional hypergeometric test implemented in the R GOstats package [47]. Enrichment of Reactome terms was evaluated using the hypergeometric test implemented in the R ReactomePA package [48], and enrichment of Disease Ontology terms was evaluated using the hypergeometric test implemented in the R DOSE package [49].

### Analysis of DNA motifs enriched in regions upstream of differentially expressed genes

DNA motifs enriched in 5000 base pair regions upstream of differentially expressed genes (DEGs) were identified using semiparametric generalized additive logistic models (GAM) [50, 51]. These analyses modeled a 1–0 indicator response variable with value 1 if a gene was identified as a DEG and value 0 if a gene had detectable expression but was not included among DEGs [50]. GAM models included two predictor variables *x*_1_ and *x*_2_, where *x*_1_ was equal to the number of motifs identified in 5000 base pair upstream regions, and *x*_2_ was equal to the length of sequence scanned excluding any coding DNA sequence [50]. For each set of DEGs evaluated, enrichment for a given motif was determined from the z-statistic and *p*-value for the *x*_1_ indicator variable [50]. To control the false discovery rate, raw *p*-values generated from among the 2935 motifs were corrected using the Benjamini-Hochberg method [42]. Analyses were replicated for a filtered dictionary containing 2935 motifs. As described previously [51], motifs within this dictionary were aggregated from a diverse set of sources including the human protein-DNA interaction database (hPDI) [52], Jaspar database [53], UniPROBE [54], TRANSFAC [55] and the ENCODE project [56, 57]. All motifs included in the dictionary had been empirically determined based upon interactions with one or more human transcription factor or unconventional DNA binding protein (e.g., ChIP-Seq, protein microarrays, SELEX technology) [51].

### Reverse transcription-polymerase chain reaction (RT-PCR)

RT-PCR was used to confirm differential expression for a subset of genes identified as differentially expressed by RNA-seq analysis. Analyses were performed using 48 h time point samples (GM6, *n* = 5; CTL, *n* = 5). PCR reactions were performed using Qiagen (Germantown, MD) primer assays (*CACNA1G*: QT00043043; *FAM65C*: QT00069671; *TMEM255A*: QT00061649), 5xAll-In-One 1st Strand cDNA Synthesis Mix (Bioland Scientific, Paramount, CA) and qPCR Master Mix (KiCqStart SYBR Green qPCR ReadyMix, Sigma, St. Louis), and cycle threshold values were generated using the iCycler iQ Detection System (Bio-Rad, Hercules, CA). Relative gene expression was evaluated using the 2^-∆∆Ct^ method [58] with normalization to heat shock protein 90 alpha family class B member 1 (*HSP90AB1*) as a housekeeping gene.

## Results

### GM6 regulates the expression of 2867 protein-coding human genes in SH-SY5Y neuroblastoma cells

**Fig. 1:**
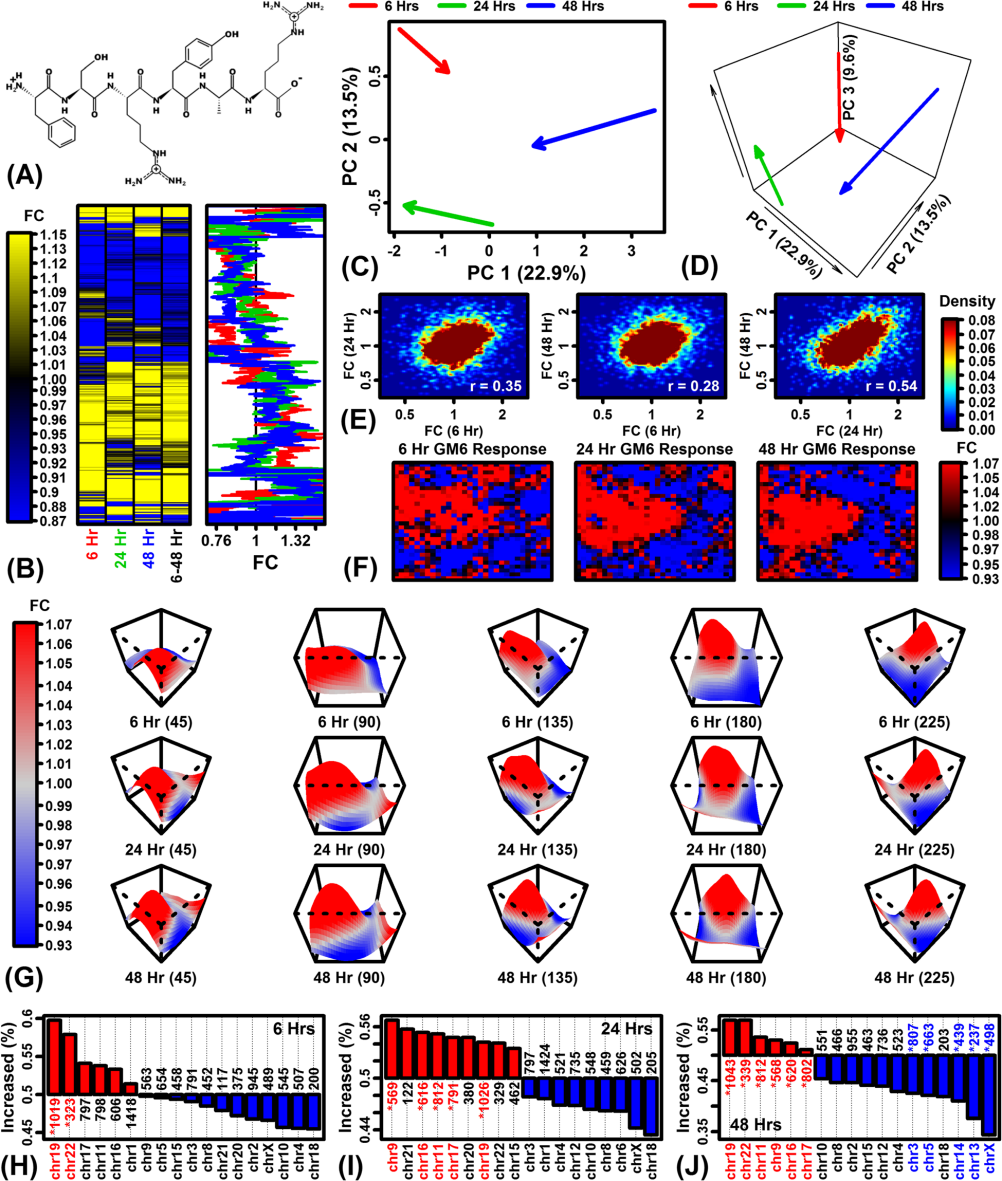
Global analysis of differential expression. (**a**) GM6 structure. (**b**) Hierarchical cluster analysis. FC estimates for 6777 protein-coding genes are shown in the yellow-blue heat map (see scale) and by lines on the right (red = 6 h; green = 24 h; blue = 48 h). Genes were clustered using average linkage and the Euclidean distance metric. The 6777 genes represent 50% of 13,554 genes included in all differential expression analyses (selected as having the largest absolute FC estimate in any of the 4 differential expression analyses). (**c**) Principal component (PC) vectors (2-dimensional). Arrows represent the effect of GM6. Arrow start points indicate the average PC coordinates of CTL samples and arrow end points indicate the average PC coordinates of GM6 samples. (**d**) PC vectors (3-dimensional). The analysis in (**c**) was repeated using the first 3 PC axes. (**e**) FC correlation between time points. Scatterplots compare FC estimates for the 3 possible pairwise time point comparisons. The color scale indicates gene density. The spearman rank correlation is indicated in the lower right for each analysis. (**f**) Self-organizing map (SOM) color images. A SOM was calculated based upon FC estimates observed for 12,562 protein-coding genes. The color scale indicates the average FC for genes assigned to each SOM region. (**g**) Smoothed SOM surfaces. The SOMs from (**f**) are alternatively represented by smoothed surfaces with the vertical axis corresponding to the average FC of genes assigned to each SOM region. Rotations are shown for each surface to provide alternative viewing angles (45, 90, 135, 180 and 225 degrees). The smoothed surface was obtained by fitting a loess function using average FC as the response variable and the other two axes as predictor variables. (**h** – **j**) Percent GM6-increased genes (FC > 1.00) per chromosome. Asterisks indicate chromosomes with a significantly large (red) or small (blue) percentage of GM6-increased genes (FDR < 0.05; Fisher’s Exact Test)

RNA-seq was used to evaluate gene expression responses of protein-coding genes to GM6 hexapeptide (Fig. 1a). Heatmap and cluster analyses showed good agreement across time points with a minority of genes exhibiting time-dependent responses (Fig. 1b). When viewed in principal component space, effects of GM6 were partially consistent at each time point with better agreement between the 24 and 48 h responses compared to the 6 h response (Fig. 1c and d). Consistent with this, expression responses were positively correlated across time points, with good agreement between 24 and 48 h responses (*r*_s_ = 0.54) but relatively weaker agreement between 6 and 48 h responses (*r*_s_ = 0.28) (Fig. 1e). Representation of global expression responses using self-organizing maps (SOMs) also showed a congruent pattern with respect to the 3 time points analyzed (Fig. 1f and g). These global analyses indicated a strong time-independent GM6 response with a comparatively minor but detectable time-dependent response. For each time point, there was a trend towards GM6-increased expression for genes located on chromosome 19 (FDR < 0.05; Figs. 1h – j). Expression of genes located on chromosomes 18 and X tended to be decreased by GM6, although this trend was significant only for chromosome X at the 48 h time point (FDR < 0.05; Fig. 1j).

Differentially expressed genes (DEGs) were next identified by comparing gene expression in GM6- and CTL-treated cells at each time point, respectively (6, 24 and 48 h; Additional file 4 A–I). To identify genes exhibiting consistent trends across time, a second differential expression analysis was also performed with samples from all time points combined into a single differential expression analysis (GM6 vs. CTL; 6–48 h; Additional file 4 J–L). The largest number of differentially expressed genes was identified with respect to the earliest (6 h) treatment time point (Table 1). Without applying a fold-change (FC) threshold, 2867 unique protein-coding genes were altered by GM6 among the 3 time points (FDR < 0.10), which included 2119 genes significantly altered in the combined 6–48 h analysis (FDR < 0.10; Table 1). When analyses were repeated with an added FC threshold (i.e., FDR < 0.10 with FC > 1.50 or FC < 0.67), 812 unique GM6-regulated genes were identified, of which 295 remained significant when samples from all time points (6–48 h) were included in the differential expression analysis (Table 1). To verify RNA-seq accuracy, we used RT-PCR to evaluate expression of 3 DEGs at the 48 h time point, including calcium voltage-gated channel subunit alpha1 G (*CACNA1G*), RIPOR family member 3 (*FAM65C*), and transmembrane protein 255A (*TMEM255A*). In each case, we observed good agreement between RNA-seq and RT-PCR results with consistent patterns of differential expression (Additional file 5).

**Table 1:** Differentially expressed genes (FDR < 0.10)

FC Threshold	Comparison	No. increased	No. decreased	Total
None	6 hours	678	480	1158
	24 hours	726	297	1023
	48 hours	446	107	553
	6–48 hours	1320	799	2119
	Pooled^a^	1724	1155	2867
FC > 1.50 or FC < 0.67	6 hours	228	115	343
	24 hours	246	36	282
	48 hours	285	29	314
	6–48 hours	265	30	295
	Pooled^a^	634	182	812

^a^Pooled: Number of genes significantly altered by GM6 (FDR < 0.10) at the indicated FC threshold in any of the 4 comparisons performed (i.e., 6, 24, 48, or 6–48 h)

The table lists the number of differentially expressed genes (DEGs) significantly altered by GM6 in SH-5YSY neuroblastoma cells. All genes were significantly altered at an FDR threshold of 0.10 and we tabulated the number of unique DEGs with and without an additional FC threshold (first column). Differential expression analyses were performed with respect to each time point (6, 24 and 48 h). We additionally identified genes with evidence for a consistent differential expression pattern with respect to each of the 3 time points (6–48 h) as well as genes differentially expressed in any of the 4 comparisons (pooled)

### Notch pathway activation is an early response to GM6 (6 h) leading to up-regulation of extracellular matrix genes (24–48 h)

**Fig. 2:**
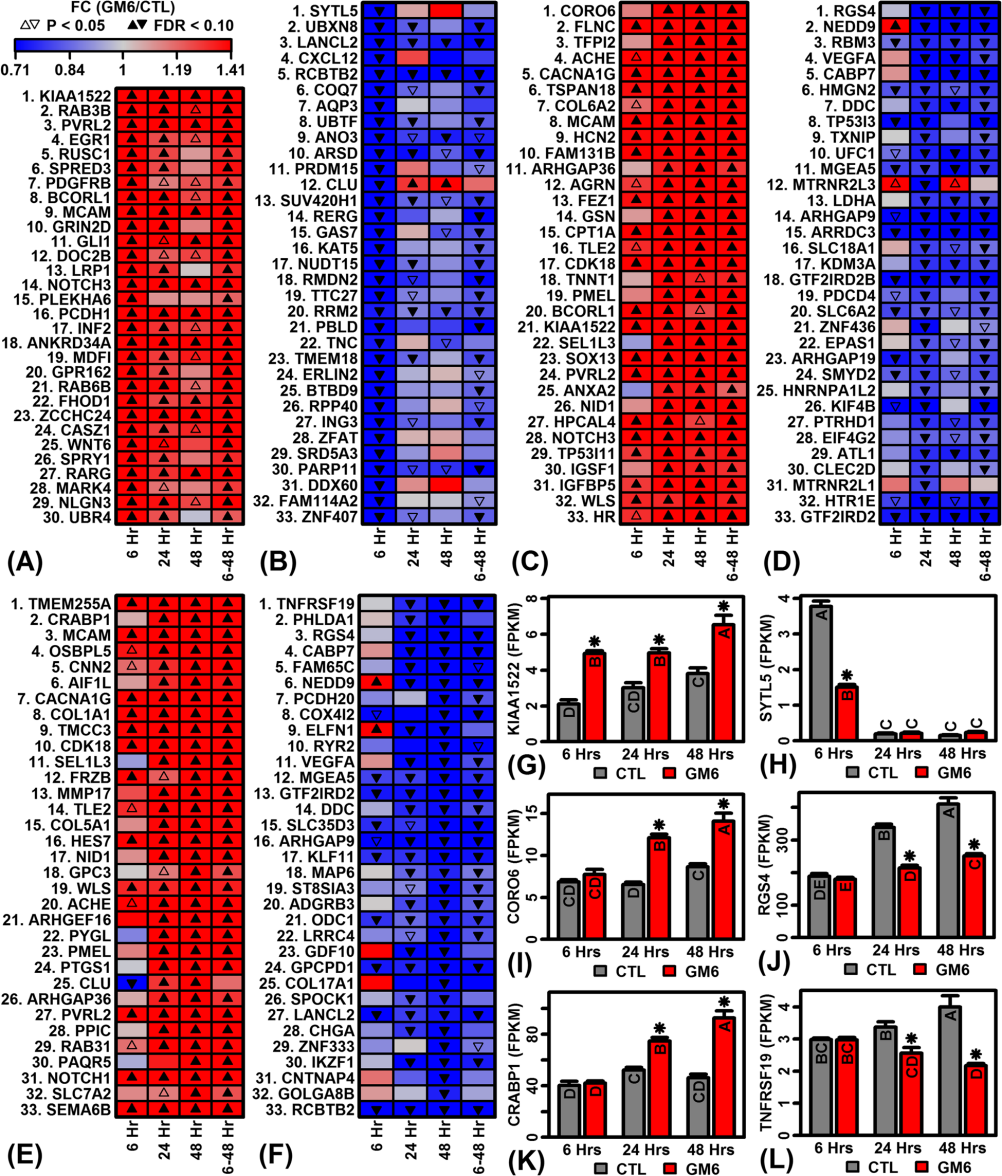
Top-ranked genes with expression most strongly altered by GM6. (**a**, **c**, **e**) GM6-increased genes. Heatmaps show genes most strongly increased by GM6 at the (**a**) 6 h, (**c**) 24 h and (**e**) 48 h time points. (**b**, **d**, **f**) GM6-decreased genes. Heatmaps show genes most strongly decreased by GM6 at the (**b**) 6 h, (**d**) 24 h and (**f**) 48 h time points. (**g**) *KIAA1522* expression. (**h**) Synaptotagmin like 5 (*SYTL5*) expression. (**i**) Coronin 6 (*CORO6*) expression. (**j**) Regulator of G protein signaling 4 (*RGS4*) expression. (**k**) Transmembrane protein 255A (*TMEM255A*) expression. (**l**) TNF receptor superfamily member 19 (*TNFRSF19*) expression. In (**g**) – (**l**), letters shown for each bar indicate results from post hoc treatment comparisons (Fisher’s least significant difference), where treatments not sharing the same letter differ significantly (*P* < 0.05)

Of genes significantly up-regulated by GM6 (FDR < 0.10), some were strongly induced with expression elevated 2–4-fold in GM6- versus CTL-treated cells (Fig. 2). Following 6 h of GM6 treatment, the most strongly up-regulated genes included *KIAA1522*, RAB3B member RAS oncogene family (*RAB3B*), and nectin cell adhesion molecule 2 (*PVRL2*) (Fig. 2a and g). Likewise, following 24 h of treatment, strongly up-regulated genes included

coronin 6 (*CORO6*), filamin C (*FLNC*) and tissue factor pathway inhibitor 2 (*TFPI2*) (Fig. 2c and i). Late GM6 responses following 48 h treatment included up-regulation of transmembrane protein 255A (*TMEM255A*), cellular retinoic acid binding protein 1 (*CRABP1*), and melanoma cell adhesion molecule (*MCAM*) (Fig. 2e and k). For most of these genes, expression responses were consistent across the 3 time points (Fig. 2a, c and e).

**Fig. 3:**
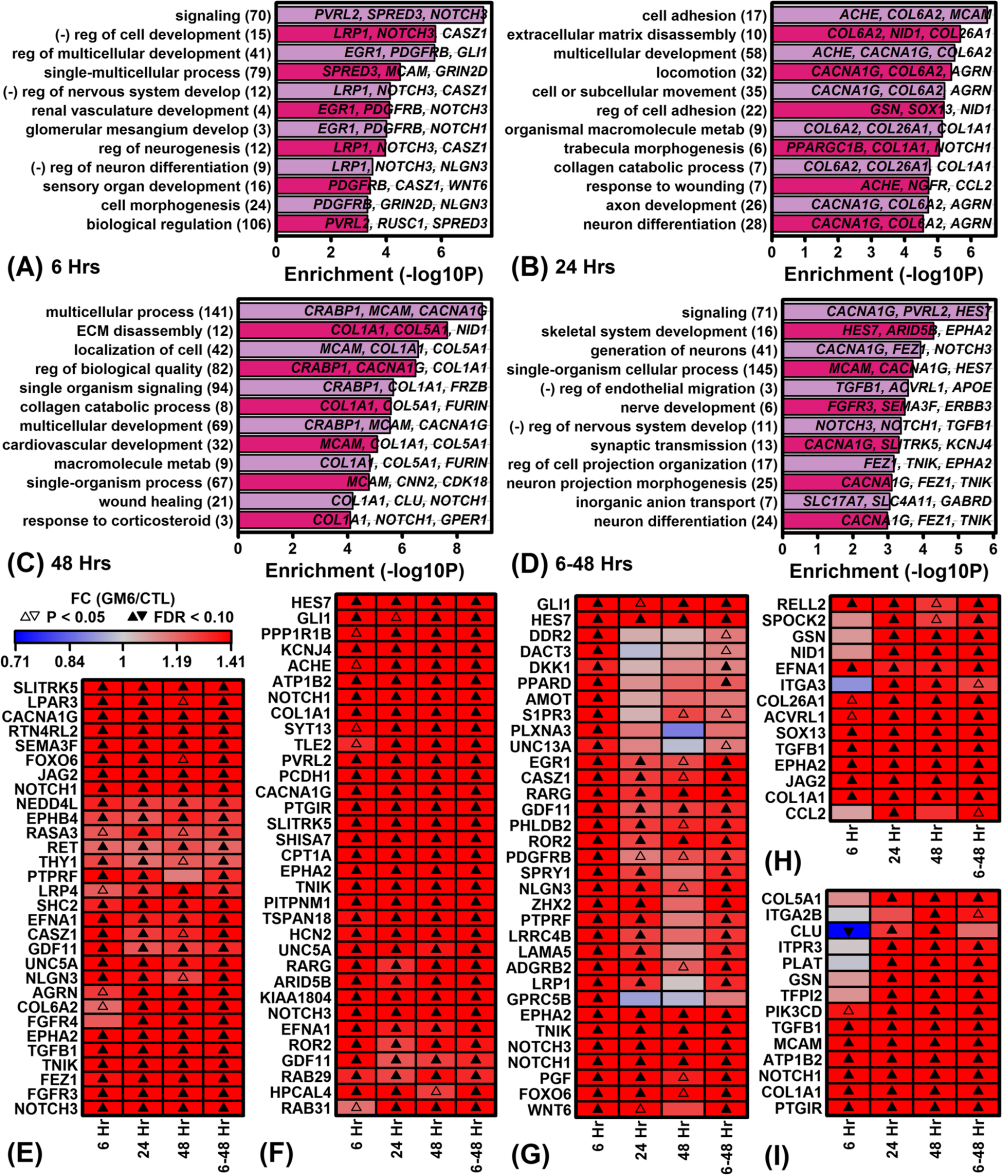
Gene Ontology (GO) biological process (BP) terms associated with GM6-increased genes. (**a** – **d**) Top-ranked GO BP terms. Figures list GO BP terms most strongly enriched with respect to the GM6-increased DEGs (FDR < 0.10, FC > 1.50) identified at (**a**) 6 h, (**b**) 24 h, (**c**) 48 h and (**d**) 6–48 h. The number of GM6-increased genes associated with each GO BP term is listed in parentheses (left margin) and exemplar genes for each term are listed in each figure. Statistical significance of enrichment (horizontal axis) was evaluated using a hypergeometric test. Labels associated with some GO BP terms are abbreviated. (**e** – **i**) Heatmaps show GM6-increased genes associated with (**e**) generation of neurons (GO:0048699), (**f**) signaling (GO:0023052), (**g**) regulation of multicellular organismal development (GO:2000026), (**h**) regulation of cell adhesion (GO:0030155) and (**i**) wound healing (GO:0042060). The genes shown for each GO BP term were most strongly increased by GM6 at (**e** – **g**) 6 h, (**h**) 24 h or (**i**) 48 h

Functional associations of genes up-regulated by GM6 were evaluated using multiple gene annotation sources, including Gene Ontology [43], KEGG [44], Reactome [45] and Disease Ontology [46] (Fig. 3 and Additional file 6). Consistent with the idea that GM6 replicates the activity spectrum of a developmental-stage protein, genes up-regulated by GM6 were linked to developmental processes and multiple aspects of neuron growth (e.g., regulation of multicellular development, regulation of neurogenesis, axon development, neuron differentiation, generation of neurons). Development-associated genes were prominently up-regulated by GM6 at the 6 h time point (Fig. 3a) and many such “early response” genes were associated with signaling (Fig. 3a), plasma membrane (Additional file 6 A) and neural-ligand receptor interaction (Additional file 6 B). Pathways associated with GM6-increased genes at 6 h included Notch, MAPK, PI3K/AKT and EGFR (Additional files 6 B and C). Up-regulated genes related to the Notch pathway encoded ligands (*JAG2*), transmembrane receptors (*NOTCH1*, *NOTCH3*) and transcriptional activating complexes (*MAML3*) (Additional file 7).

Whereas genes up-regulated by GM6 at 6 h were associated with signaling and the plasma membrane, genes up-regulated at 24 and 48 h were most frequently associated with cell adhesion, extracellular matrix (ECM) and wound healing (Fig. 3b and c). Consistent with this, genes up-regulated by GM6 following 24 and 48 h of treatment had structural functions related to collagen trimer, collagen formation and collagen fibril assembly (Fig. 3b, c, Additional file 6 E–L). Although fewer “late response” up-regulated genes were associated with signaling, genes increased at 24 and 48 h were associated with the calcium signaling and MAPK pathways (Additional file 6 F). Among genes increased by GM6 in the combined differential expression analysis (6–48 h), there was additionally significant enrichment with respect to the hedgehog signaling pathway (Additional file 6 N). Up-regulated genes related to the hedgehog pathway encoded ligands (*DHH*), kinases (*CSNK1G2*), transcription factors (*GLI1*, *GLI2*) and target genes (*CCND1*) (Additional file 8).

### GM6 represses the expression of genes linked to inflammation, mitochondria, mRNA processing and chromatin organization

Genes most strongly down-regulated by GM6 included synaptotagmin like 5 (*SYTL5*), regulator of G protein signaling 4 (*RGS4*), and TNF receptor superfamily member 19 (*TNFRSF19*) following 6, 24 and 48 h of treatment, respectively (Fig. 2b, d, f, h, j and l). Among down-regulated genes, there was less consistency across time points, particularly comparing the early 6 h to the late 24 and 48 h responses (Fig. 2b, d and f). For some genes, significant and opposite responses were observed at different time points, e.g., clusterin (*CLU*), neural precursor cell expressed developmentally down-regulated 9 (*NEDD9*), MT-RNR2 like 3 (*MTRNR2L3*) and extracellular leucine rich repeat and fibronectin type III domain containing 1 (*ELFN1*) (Fig. 2b, d and f).

**Fig. 4:**
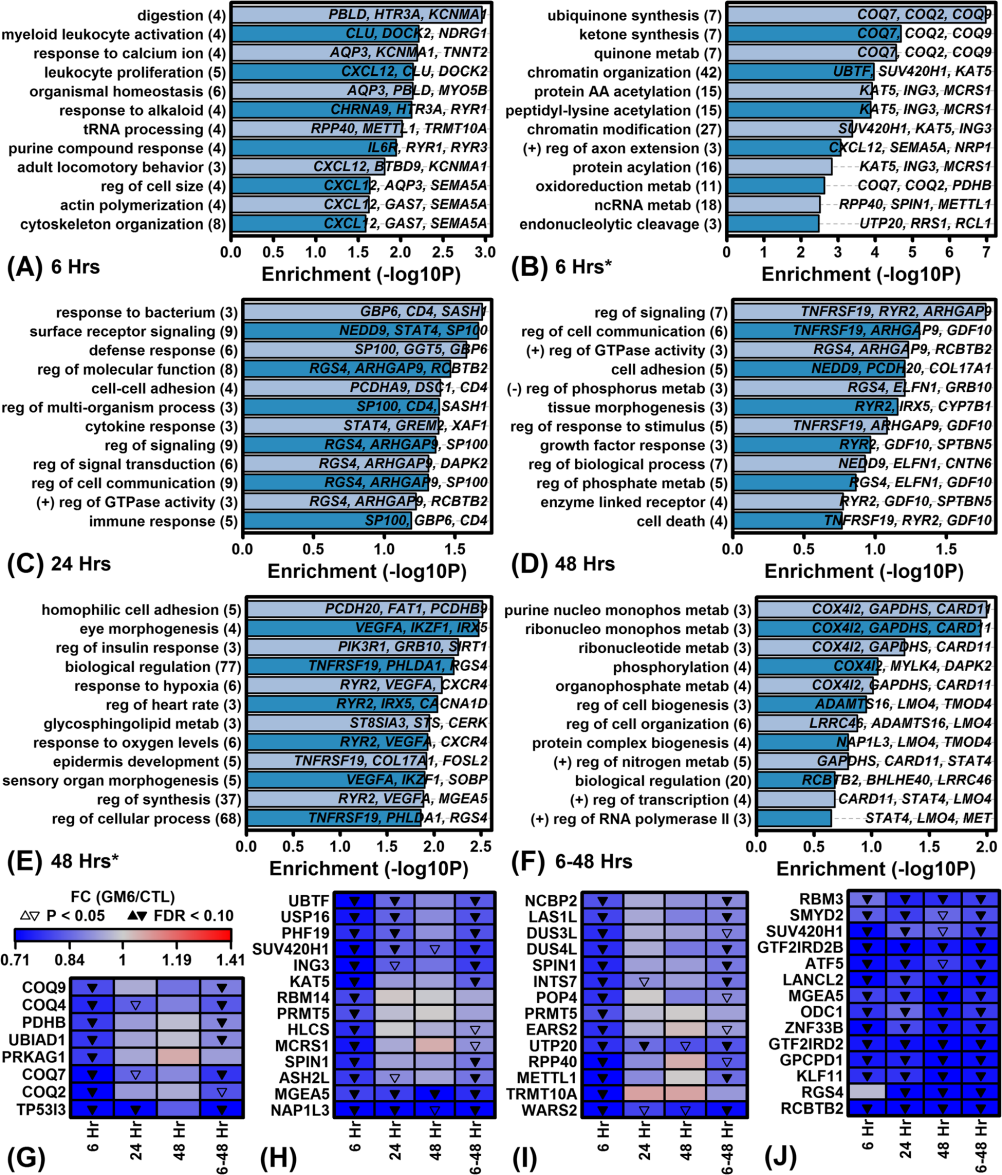
Gene Ontology (GO) biological process (BP) terms associated with GM6-decreased genes. (**a** – **f**) Top-ranked GO BP terms. Figures list GO BP terms most strongly enriched with respect to the GM6-decreased DEGs identified at (**a**, **b**) 6 h, (**c**) 24 h, (**d**, E) 48 h and (**f**) 6–48 h. The analyzed DEGs were significant at the threshold of (**a**, **c**, **d**, **f**) FDR < 0.10 and FC < 0.67 or the less stringent threshold of (**b**, **e**) FDR < 0.10 and FC < 1.00 (*). The number of GM6-decreased genes associated with each GO BP term is listed in parentheses (left margin) and exemplar genes for each term are listed in each figure. Statistical significance of enrichment (horizontal axis) was evaluated using a hypergeometric test. Labels associated with some GO BP terms are abbreviated. (**g** – **j**) Heatmaps show GM6-decreased genes associated with (**g**) oxidoreduction coenzyme metabolic process (GO:0006733), (**h**) chromatin organization (GO:0006325), (**i**) ncRNA metabolic process (GO:0034660) and (**j**) biological regulation (GO:0065007). Genes shown for each GO BP term were most strongly decreased by GM6 at 6 h

At the early 6 h time point, GM6 decreased expression of genes associated with the synthesis and metabolism of the mitochondrial coenzyme ubiquinone (e.g., *COQ2*, *COQ7*, *COQ9*; Fig. 4b). At multiple time points, genes down-regulated by GM6 were frequently associated with immunological functions (e.g., myeloid leukocyte activation, leukocyte proliferation, response to bacterium, defense response, cytokine response, immune response and cytokine receptor interaction; Figs. 4 and Additional file 9). Genes decreased by GM6 were frequently associated with mRNA processing or transcription (e.g., spliceosome, RNA transport, RNA degradation, tRNA processing, processing of pre-mRNA, transcription) as well as processes related to 3-dimensional chromatin structure (e.g., chromatin organization/modification, protein acylation) (Additional file 9). Interestingly, while GM6 decreased expression of genes associated with cell death (Fig. 4d), GM6 also down-regulated the expression of cell cycle-associated genes involved in diseases of proliferation and cancer (Additional file 9 F and H). The specific types of cancer associated with GM6-decreased genes included stomach cancer, neuroblastoma, lung cancer, pancreatic cancer, glioblastoma multiforme, astrocytoma and skeletal muscle cancer (Additional file 9). Specific signaling pathways linked to GM6-decreased genes included mTOR, VEGF and Fc epsilon RI (Additional file 9 J: Figure).

### GM6-increased genes are associated with GC-rich C2H2 zinc finger motifs and decreased genes are associated with AT-rich helix-turn-helix homeodomain motifs

**Fig. 5:**
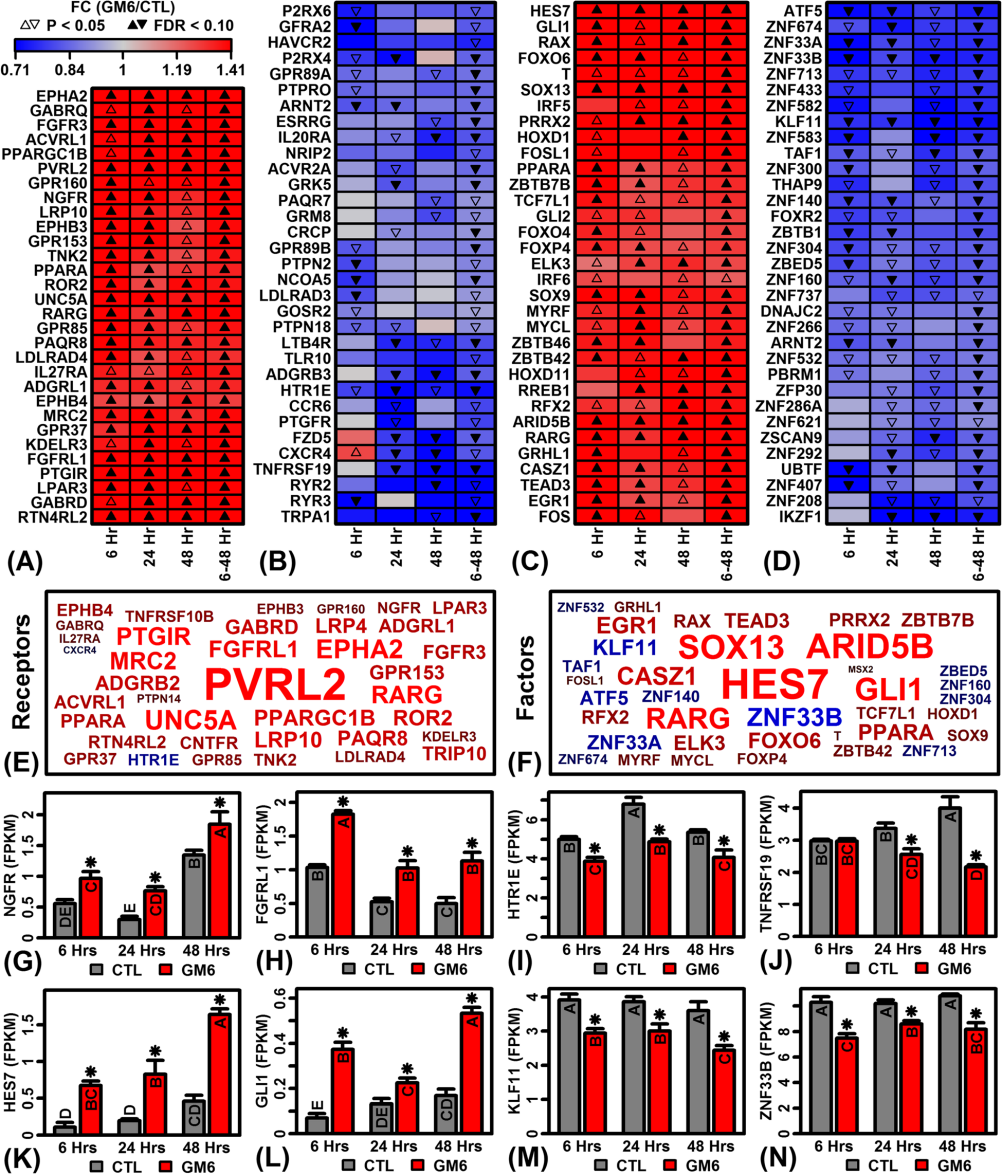
GM6-regulated genes encoding receptors and TFs. (**a**) Receptor-encoding genes most consistently up-regulated by GM6 across the 3 time points (6–48 h). (**b**) Receptor-encoding genes most consistently down-regulated by GM6 across the 3 time points (6–48 h). (**c**) TF-encoding genes most consistently up-regulated by GM6 across the 3 time points (6–48 h). (**d**) TF-encoding genes most consistently down-regulated by GM6 across the 3 time points (6–48 h). (**e**, **f**) Gene symbol clouds for genes encoding (**e**) receptors and (**f**) transcription factors. The size of each symbol corresponds to the significance of *p*-values across the three time points (6, 24 and 48 h; red = GM6-increased; blue = GM6-decreased). (**g**) Nerve growth factor receptor (*NGFR*) expression. (**h**) Fibroblast growth factor receptor like 1 (*FGFRL1*) expression. (**i**) 5-hydroxytryptamine receptor 1E (*HTR1E*) expression. (**j**) TNF receptor superfamily member 19 (*TNFRSF19*) expression. (**k**) Hes family bHLH transcription factor 7 (*HES7*) expression. (L) GLI family zinc finger 1 (*GLI1*) expression. (**m**) Kruppel like factor 11 (*KLF11*) expression. (**n**) Zinc finger protein 33B (*ZNF33B*) expression. In (**g**) – (**n**), letters shown for each bar indicate results from post hoc treatment comparisons (Fisher’s least significant difference), where treatments not sharing the same letter differ significantly (*P* < 0.05)

Our findings suggested that GM6 has diverse effects on neurogenesis, collagen synthesis and immune/inflammatory processes. We hypothesized that these effects are mediated by signaling pathways linked to multiple receptors and DNA transcription factors. We therefore evaluated effects of GM6 on the expression of genes encoding extracellular or intracellular receptors (Fig. 5a and b). At multiple time points, GM6 increased expression of nerve growth factor receptor (*NGFR*) and fibroblast growth factor receptor like 1 (*FGFRL1*) (Fig. 5a, e and f) and decreased expression of 5-hydroxytryptamine receptor 1E (*HTR1E*) and TNF receptor superfamily member 19 (*TNFRSF19*) (Fig. 5b, g and h). Likewise, genes encoding transcription factors were altered by GM6 at multiple time points (Fig. 5c and d). Among these, the most strongly increased by GM6 included hes family bHLH transcription factor 7 (*HES7*) and GLI family zinc finger 1 (*GLI1*) (Fig. 5i and j), while the most strongly decreased included KruppelAdd like factor 11 (*KLF11*) and zinc finger protein 33B (*ZNF33B*) (Fig. 5k and l).

To characterize DNA binding sites interacting with such factors, we screened 2935 DNA motifs to identify those significantly enriched in regions upstream of GM6-regulated genes. Each of the 2935 motifs had been empirically determined based upon interactions with a mammalian transcription factor or unconventional DNA binding protein (uDBPs) [51]. At each time point, this identified > 400 DNA motifs enriched with respect to sequences upstream of GM6-increased genes, with fewer motifs enriched with respect to GM6-decreased genes (FDR < 0.05; Additional file 10). A clear pattern was that GM6-increased genes were associated with motifs having high GC content (Additional file 11), whereas GM6-decreased genes were associated with motifs having high AT content (Additional file 12). A motif with consensus sequence 5-GAGGG/CCCTC-3 and known to interact with zinc finger DHHC-type containing 5 (*ZDHHC5*) was either the top or second most highly ranked motif associated with GM6-increased genes at each time point (Additional file 11). For GM6-decreased genes at 6 and 24 h, a motif with consensus 5-TTGCAA/TTGCAA-3 and interacting with GIT ArfGAP 2 (GIT2) was among the most enriched in upstream regions (Additional file 12 A and B). In several cases, motifs upstream of GM6-increased genes were known to interact with proteins encoded by mRNAs for which expression was altered by GM6 (Additional file 13 A and B), such as signal transducer and activator of transcription 3 (*STAT3*), zinc finger matrin-type 2 (*ZMAT2*), annexin A11 (*ANXA11*) and MYCN proto-oncogene bHLH transcription factor (*MYCN*) (Additional file 13 C–F). Likewise, some motifs upstream of GM6-decreased genes interacted with proteins encoded by mRNAs down-regulated by GM6, including EEF1A lysine methyltransferase 3 (*METTL21B*), homeobox D11 (*HOXD11*), thyroid hormone receptor interactor 10 (*TRIP10*) and CUGBP Elav-like family member 5 (*CELF5*) (Additional file 13 G–J).

**Fig. 6:**
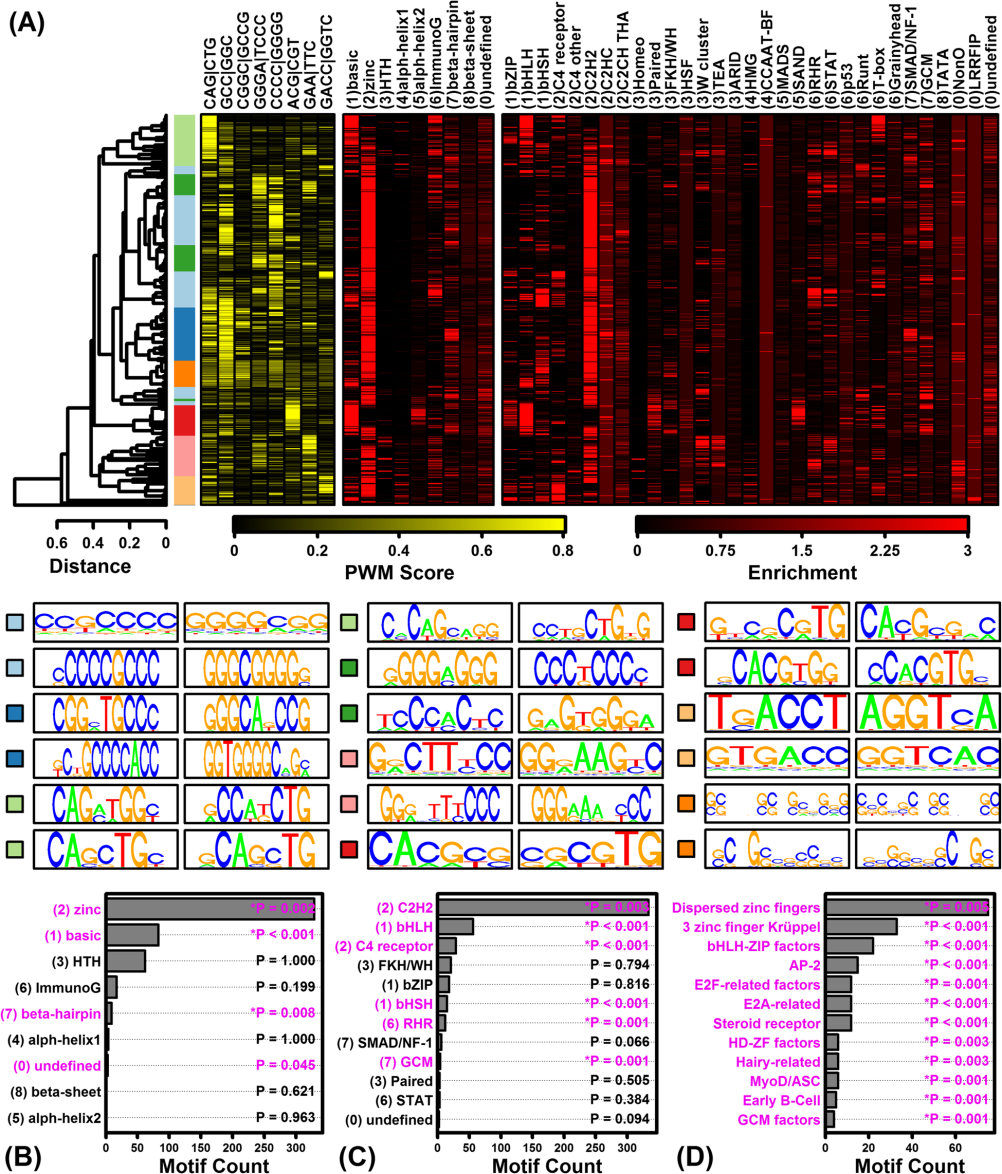
Variation and trends among DNA motifs enriched with respect to GM6-increased genes (6–48 h). (**a**) Motif cluster analysis. The 656 motifs significantly enriched in regions upstream of GM6-increased genes (6–48 h, FDR < 0.05) were clustered based upon scores assigned to each position weight matrix (PWM). Scores were calculated based upon the degree of correspondence between each PWM and a series of short *k*-mer sequences (yellow-black heatmap). The 656 motifs were divided into groups based upon the cluster analysis, and representative sequence logos for each group are shown below the heatmaps (see color scale). Black-red heatmaps show enrichment scores representing the degree to which each motif resembles those from various TF superfamilies and classes. For each motif, enrichment scores represent log10-transformed p-values derived from the test of whether motifs belonging to a given TF superfamily or class are overrepresented among the set of 300 similar “nearest neighbor” motifs (Fisher’s Exact Test). (**b**) TF superfamilies most enriched among the 656 motifs. (**c**) TF classes most enriched among the 656 motifs. (**d**) TF families most enriched among the 656 motifs

**Fig. 7:**
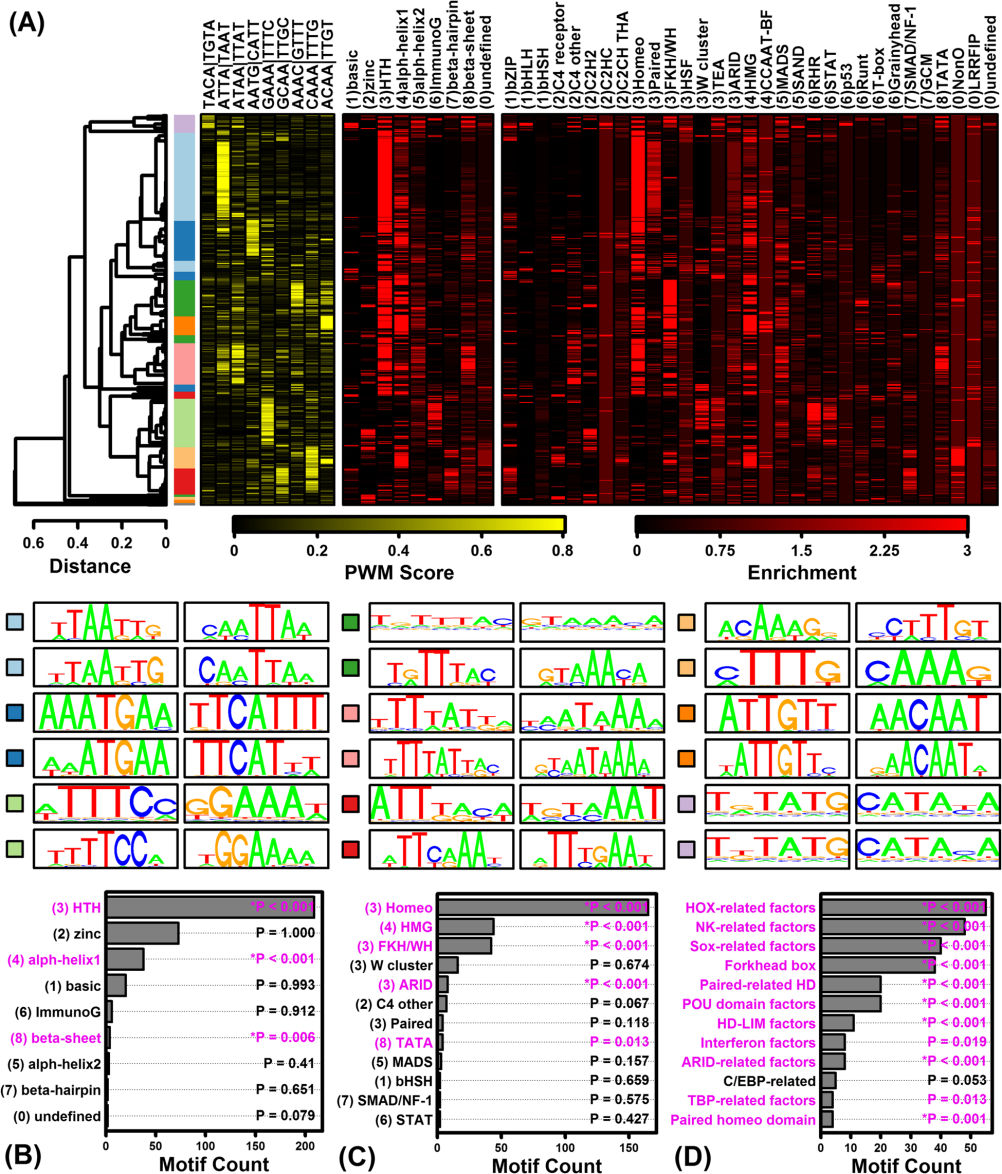
Variation and trends among DNA motifs enriched with respect to GM6-decreased genes (6–48 h). (**a**) Motif cluster analysis. The 498 motifs significantly enriched in regions upstream of GM6-decreased genes (6–48 h, FDR < 0.05) were clustered based upon scores assigned to each position weight matrix (PWM). Scores were calculated based upon the degree of correspondence between each PWM and a series of short *k*-mer sequences (yellow-black heatmap). The 498 motifs were divided into groups based upon the cluster analysis, and representative sequence logos for each group are shown below the heatmaps (see color scale). Black-red heatmaps show enrichment scores representing the degree to which each motif resembles those from various TF superfamilies and classes. For each motif, enrichment scores represent log10-transformed p-values derived from the test of whether motifs belonging to a given TF superfamily or class are overrepresented among the set of 300 similar “nearest neighbor” motifs (Fisher’s Exact Test). (**b**) TF superfamilies most enriched among the 498 motifs. (**c**) TF classes most enriched among the 498 motifs. (**d**) TF families most enriched among the 498 motifs

Considering genes altered by GM6 in the combined analyses (6–48 h), a large number of motifs were significantly enriched in upstream regions of both GM6-increased and GM6-decreased genes (GM6-increased: 656 motifs; GM6-decreased: 498 motifs; FDR < 0.05; Additional file 10). As noted above, motifs associated with GM6-increased genes were GC-rich, whereas motifs associated with GM6-decreased genes were AT-rich (Additional files 11, 12, 13). Consistent with this, the motifs were also associated with different transcription factor superfamilies, classes and families (Figs. 6a and 7a) [59]. Motifs associated with GM6-increased genes were most strongly associated with TFs from the C2H2 class and zinc-coordinating DNA-binding domain superfamily (Fig. 6b and c), including many motifs belonging to the dispersed zinc finger, 3 zinc finger Kruppel or bHLH-ZIP factor families (Fig. 6d). In contrast, motifs associated with GM6-decreased genes were most strongly associated with TFs from the helix-turn-helix domain superfamily and homeo class (Fig. 7b and c), with many motifs belonging to the HOX-, NK- and SOX-related families (Fig. 7d).

### GM6 regulates the expression of ALS-associated genes involved in neurogenesis, axon guidance and the intrinsic apoptosis pathway

**Fig. 8:**
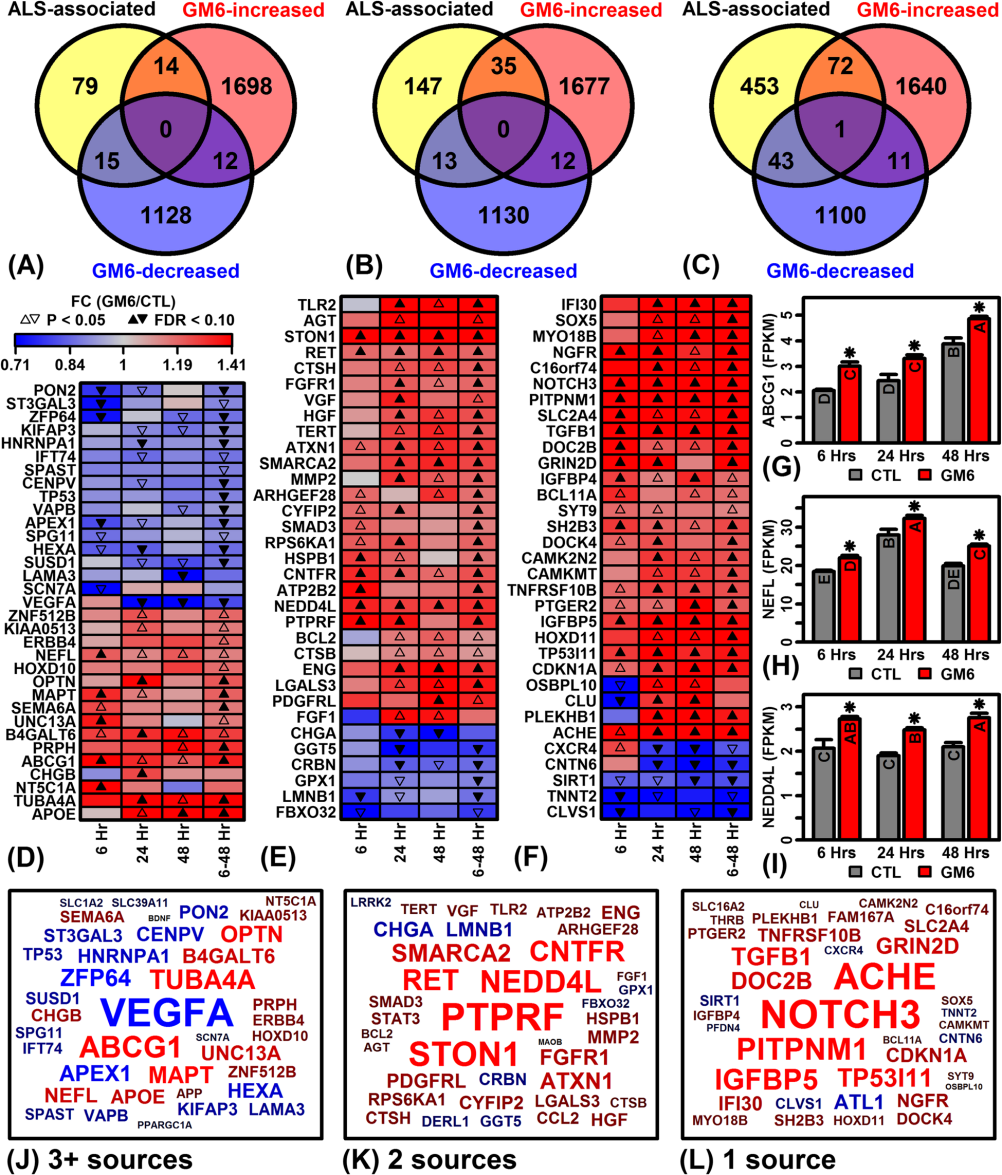
ALS-associated genes regulated by GM6. (**a**) ALS-associated genes (3+ database sources) overlap with GM6-increased/decreased genes (FDR < 0.10). (**b**) ALS-associated genes (2 database sources) overlap with GM6-increased/decreased genes (FDR < 0.10). (**c**) ALS-associated genes (1 database source) overlap with GM6-increased/decreased genes (FDR < 0.10). In (**a**) – (**c**), GM6-increased genes include those increased by GM6 with respect to any of the 4 differential expression analyses (6, 24, 48, and/or 6–48 h; FDR < 0.10), and GM6-decreased genes include those decreased by GM6 with respect to any of the 4 differential expression analyses (6, 24, 48, and/or 6–48 h; FDR < 0.10). (**d**) ALS-associated genes (3+ sources). (E) ALS-associated genes (2 sources). (**f**) ALS-associated genes (1 source). In (D) – (**f**), heatmaps show the ALS-associated genes most consistently altered by GM6 (6–48 h). (**g**) ATP binding cassette subfamily G member 1 (*ABCG1*) expression. (**h**) Neurofilament light (*NEFL*) expression. (**i**) Neural precursor cell expressed developmentally down-regulated 4-like E3 ubiquitin protein ligase (*NEDD4L*) expression. In (**g**) – (**i**), letters shown for each bar indicate results from post hoc treatment comparisons (Fisher’s least significant difference), where treatments not sharing the same letter differ significantly (*P* < 0.05). (**j**) Gene symbol cloud for ALS-associated genes (3+ sources). (**k**) Gene symbol cloud for ALS-associated genes (2 sources). (**l**) Gene symbol cloud for ALS-associated genes (1 source). In (**j**) – (**l**), the size of each symbol corresponds to the significance of p-values across the three time points (6, 24 and 48 h; red = GM6-increased; blue = GM6-decreased)

We hypothesized that GM6 may regulate the expression of genes associated with ALS. We identified ALS-associated genes from 9 database sources (Additional file 14 and Fig. 8) [43, 44, 46, 60–66]. Among the 9 sources, 108 genes were common to 3 or more databases and expressed at levels sufficient to be included in differential expression analyses. Of these 108 ALS-associated genes, expression of 29 (26.9%) was significantly altered by GM6 with respect to one or more time points, including 14 GM6-increased genes (FDR < 0.10) and 15 GM6-decreased genes (FDR < 0.10) (Fig. 8a, d and j). The overlap between the 108 ALS-associated genes and GM6-increased genes was non-significant (*P* = 0.27); however, overlap between ALS-associated genes and GM6-decreased genes was significant (*P* = 9.38 × 10^− 3^) (Fisher’s Exact test). Genes robustly associated with ALS through 3 or more database sources thus overlapped significantly with GM6-down-regulated genes (Fig. 8a, d and j). Among such genes, we identified 3 that were at least marginally up-regulated (*P* < 0.05) at all time points (Fig. 8d), including *B4GALT6*, *ABCG1* (Fig. 8g), and *NEFL* (Fig. 8h).

These analyses were repeated with respect to genes linked to ALS less robustly, i.e., based upon 1 or 2 of the databases included in our analysis (Fig. 8b, c, e, f, k and l). Among 195 genes linked to ALS by 2 databases and expressed in our experiments, expression of 48 (24.6%) had been significantly altered by GM6 (Fig. 8b, e and k). The overlap was significant with respect to GM6-increased genes (*P* = 3.30 × 10^− 3^) but not with respect to GM6-decreased genes (*P* = 0.66) (Fisher’s Exact Test). We identified 3 such genes significantly elevated by GM6 at all time points (Fig. 8e), including *STON1*, *RET* and *NEDD4L* (Fig. 8i). Among 569 genes linked to ALS by 1 database and expressed in our experiments, expression of 116 (20.3%) had been significantly altered by GM6 (Fig. 8c, f and l). Several of these genes were significantly altered by GM6 at all time points (e.g., *NOTCH3*, *TGFB1*, *IGFBP5*), although the observed overlap was non-significant with respect to GM6-increased and GM6-decreased genes (*P* ≥ 0.17; Fisher’s Exact Test). These analyses show that genes most robustly linked to GM6 (2 or 3+ database sources) overlap significantly with GM6-regulated genes identified by our analyses.

We next used gene annotation databases [43–45] to better understand functions of ALS-associated genes (2+ sources) significantly regulated by GM6 at one or more time points (FDR < 0.10) (Additional file 15). ALS-associated genes up-regulated by GM6 were associated with developmental biology, regulation of nervous system development, negative regulation of apoptosis, positive regulation of neurogenesis, axon development/guidance, neuron projection, and intermediate filament cytoskeleton (Additional file 15 A, C, E and F). ALS-associated genes down-regulated by GM6 were associated with apoptosis, intrinsic pathway for apoptosis, regulation of programmed cell death, apoptotic mitochondrial changes, mitochondrial matrix, positive regulation of chromatin modification and morphology modification (Additional file 15 B, D, F and G).

## Discussion

ALS is a debilitating and ultimately fatal neurodegenerative disease for which few treatment options are currently available. The peptide drug GM604 (GM6 or Alirinetide) has been developed as a multi-target candidate ALS therapeutic with pharmacokinetic properties similar to other small molecule drugs entering the central nervous system [10]. This study used RNA-seq to provide the first complete analysis of gene expression responses to GM6, with the purpose of developing hypotheses regarding mechanisms of action. Our findings demonstrate that GM6 significantly alters the expression of > 2800 protein-coding genes in SH-SY5Y neuroblastoma cells, leading to expression responses consistent with activation of multiple neurodevelopmental signaling pathways (e.g., Notch and Hedgehog), increased abundance of proteins contributing to the extracellular matrix or cell adhesion (*COL1A1*, *COL6A2*, *COL26A1*), and modulation of chromatin structure and a network of transcription factors interacting with *cis*-regulatory elements (*STAT3*, *HOXD11*, *HES7*, *GLI1*). We identified 77 genes linked to ALS by multiple database sources that were also regulated by GM6 at one or more time points (e.g., *TUBA4A, NEFL, NEDD4L, FGFR1, RET*). Taken together, our findings support the hypothesis that GM6 enables neuron survival by restoring an embryonic-stage gene expression program [19], while additionally strengthening cell adhesion and an extracellular matrix scaffold supporting the central and peripheral nervous systems [67, 68]. This multi-target mechanism of action is unique among existing ALS drug candidates and may provide therapeutic benefit for ALS and multiple other diseases characterized by progressive neuron loss (e.g., Alzheimer’s, Huntington’s and Parkinson’s diseases) [20].

In recent decades, more than 50 ALS randomized controlled trials have been performed, but the majority of these have failed to provide evidence of efficacy for the investigational product [5, 69, 70]. This lack of progress may be attributed, at least in part, to the multifactorial nature of ALS [71] and the limitations of drugs designed to narrowly target a single protein or cellular pathway [20, 72]. In recognition of this, the peptide drug GM6 was not designed to regulate any one specific pathway, but instead was developed to mimic the activity spectrum of a neurotrophic factor expressed during embryological development [10, 19]. Consistent with this, our RNA-seq findings showed that GM6 increased expression genes belonging to the Notch (*JAG2*, *NOTCH1*, *NOTCH3*) and hedgehog (*GLI1*, *DHH*) neurodevelopmental pathways. Both pathways are critical mediators of neurodevelopment with roles in morphogenesis, cell-cell signaling, proliferation, differentiation and apoptosis [73]. The contributions of Notch and hedgehog to ALS pathophysiology remains unclear. One study demonstrated increased abundance of Notch proteins in spinal cords from SOD1 G93A transgenic mice and NSC34 cells transfected with mutant SOD1 [74]. However, hedgehog activation is cytoprotective against oxidative stress [75–77], and hedgehog activity is repressed in CSF from ALS patients [78]. While GM6 may activate Notch and hedgehog independently, we favor a model involving Notch-hedgehog crosstalk, in which GM6 up-regulates Notch ligand and receptors with secondary activation of hedgehog. This Notch-hedgehog axis was recently supported by experiments showing declines in hedgehog signaling following Notch inhibition [79]. Interestingly, this same study demonstrated that Notch intracellular domain expression is decreased in spinal cord motor neurons from transgenic mutant SOD1 mice, with the loss of intracellular domain expression correlating with the onset of disease symptoms [79]. Concurrent with this, motor neurons from mutant SOD1 mice exhibited decreased expression of Gli family zinc fingers *Gli2* and *Gli3* [79]. These studies suggest that mutant SOD1 decreases activation of a Notch-hedgehog axis in motor neurons, potentially indicating a mechanism contributing to ALS pathogenesis. Our current data indicate that this effect may be countered by GM6 treatment through the up-regulation of ligands, receptors and transcription factors associated with the Notch and hedgehog pathways (Additional files 7 and 8).

The extracellular matrix provides a scaffold and microenvironment that supports neurons and has an active role in directing axon extension and growth [67, 68]. An unexpected finding from this study was that prolonged GM6 treatment (24–48 h) increased expression of genes encoding collagen (*COL1A1*, *COL6A2*, *COL26A1*) and other proteins localized to the ECM or functioning in cell adhesion (*TFIP2*, *MMP17*, *AGRN*, *MCAM*). Potentially, increased expression of such genes by GM6 may have been mediated by up-regulation of transforming growth factor beta 1 (*TGFB1*), which was significantly increased by GM6 at all time points (Fig. 8f) examined and is a positive regulator of collagen synthesis [80]. These effects on the expression of genes encoding structural proteins, including many localized to skin, may be expected under the hypothesis that GM6 signals through developmental pathways that may be active prior differentiation of neural and integumentary lineages from ectoderm. The importance of ECM proteins in ALS is not yet established, although an ECM abnormality was suggested decades ago by the observation that ALS patients do not develop bedsores as expected in immobilized patients [81]. Since then, numerous studies have found abnormal collagen and metalloproteinase levels in the skin and spinal cord from ALS patients [82, 83], with some studies demonstrating reduced collagen content and smaller collagen fibrils in the skin and spinal cord [84, 85], whereas other studies have demonstrated increased dermal collagen in the sacral region [86]. These and other findings have supported an ECM abnormality affecting multiple tissues in ALS patients, although the contribution of this to the disease pathogenesis is not understood [82, 83]. Nonetheless, the up-regulation of genes contributing to ECM and cell adhesion proteins was a significant effect of GM6 in the current study, which would be expected to bolster the scaffold supporting axon growth [67, 68] or may otherwise influence underlying collagen metabolism deficits in ALS patients [84, 85].

Signaling pathways activated or inhibited by GM6 exert their effects by actively modulating downstream gene transcription. This regulation can occur at multiple levels through epigenetic modifications of chromatin architecture to shift the euchromatin-heterochromatin balance [87, 88], and through the control of transcription factors interacting with *cis*-regulatory elements in a sequence-specific fashion [89]. Our results provide evidence that GM6 may alter gene transcription through both mechanisms. Genes consistently down-regulated by GM6 were frequently localized to the nucleus (Additional file 9 M) and known to function in chromatin organization and protein acetylation (Fig. 4b). We additionally noted a strong pattern in which regions upstream of GM6-increased genes were enriched with GC-rich motifs interacting with zinc C2H2 transcription factors, whereas regions upstream of GM6-decreased genes were enriched with AT-rich motifs interacting with helix-turn-helix homeodomain transcription factors (Figs. 6, 7; Additional files 11 and 12). These trends may indicate that GM6 partially regulates gene expression through epigenetic mechanisms and in particular by controlling methylation status of CpG islands in promoters of GM6-increased genes identified by our analysis [90]. Notably, for instance, several genes down-regulated by GM6 encoded methyltransferases such as *METTL21B* (also known as *FAM119B* and *EEF1AKMT3*) and lysine methyltransferase 5B (*SUV420H1*). Enrichment of GC-rich motifs upstream of GM6-increased genes may also reflect modulation of transcription factors targeting GC-rich motifs (e.g., *STAT3*, *ZMAT2*, *MYCN*), whereas enrichment of AT-rich motifs upstream of GM6-decreased genes may reflect modulation of factors targeting AT-rich motifs (e.g., *HOXD11*, *TRIP10*, *CEL5F*). Altogether, our findings provide evidence for a diverse network of transcription factors regulated by GM6, including developmental transcription factors associated with Notch (*HES7*) (Fig. 5i) and hedgehog signaling (*GLI1*, *GLI2*) (Fig. 5c). Regulation of this network combined with epigenetic modifications of chromatin structure likely contribute to the large number of GM6-regulated genes identified by our study, consistent with the diverse modes of gene regulation known to mediate neurodevelopment [87, 88].

We investigated effects of GM6 on the expression of genes previously associated with ALS to identify disease-relevant processes and pathways altered as part of the transcriptional response (Fig. 8). ALS-associated genes altered by GM6 were functionally consistent with other genes identified in our analysis and frequently related to neurogenesis, axon guidance and the intrinsic apoptosis pathway (Additional file 15). Up-regulated genes related to neurogenesis included neural precursor cell expressed developmentally down-regulated 4-like E3 ubiquitin protein ligase (*NEDD4L*), fibroblast growth factor receptor 1 (*FGFR1*), and ret. proto-oncogene (*RET*). *NEDD4L* encodes a HECT domain E3 ubiquitin ligase expressed in mouse embryonic neurons undergoing proliferation and migration [91]. Similarly, *FGFR1* encodes a member of the fibroblast growth factor receptor family that functions in neuron migration [92] and is highly expressed in the hippocampus as well as astrocytes and oligodendrocytes [93]. *RET* encodes a tyrosine protein kinase family transmembrane receptor essential for development of the enteric nervous system [94] and has been found to mediate neurite extension in SH-SY5Y cells [95]. Several ALS-associated genes regulated by GM6 were associated with microtubule stability (*TUBA4A* and *NEFL*), which is thought to be a key factor underlying disease susceptibility and a promising avenue for development of new ALS therapies [96]. For example, *TUBA4A* has recently been associated with ALS by genetic studies [97] and encodes an alpha-tubulin protein integral to the microtubule cytoskeleton and neuronal architecture [98]. Likewise, *NEFL* encodes a neurofilament protein that contributes to microtubule cytoskeleton organization and axonal transport (retrograde and anterograde). The regulation of these and other ALS-associated genes by GM6 supports the hypothesis that the drug is able to alter pathways involved in the disease process and provides direction for future translational studies.

This study used RNA-seq to provide the first complete characterization of the transcriptional response to GM6 in the SH-SY5Y neuroblastoma cell line. We chose to work with SH-SY5Y cells because they provide a well-characterized model system frequently used in mechanistic studies of ALS and other neurodegenerative diseases [24–29]. Since these cells have a catecholaminergic phenotype, they are especially well-suited for studies of diseases in which dopaminergic cell death plays a central role in disease pathology (e.g., Parkinson’s disease) [25]. We expect that transcriptional responses here observed in SH-SY5Y cells are, to some degree, representative of those that would occur in diverse neuronal cell types [24]. However, it should be noted that SH-SY5Y cells are not motor neurons, which are the key cell type that is lost in ALS to drive disease progression [1, 3]. In future work, therefore, it will be valuable to confirm our findings using other in vitro models expected to reflect motor neuron physiology more faithfully, such as NSC-34 cells [99], primary motor neurons [100], or stem cell-derived motor neurons [101]. Finally, although many genes were identified as differentially expressed in our study, confirmation of RNA-seq findings with RT-PCR was performed only for a selected subset of genes (i.e., *CACNA1G*, *FAM65C* and *TMEM255A*). The agreement between RNA-seq and RT-PCR results (Additional file 5) provides assurance that our findings are robust, but it will nonetheless be valuable in future work to provide similar RT-PCR confirmation for other genes identified by our analysis [102].

The purpose of this study was not to evaluate efficacy of GM6 as an ALS treatment, but rather to develop hypotheses regarding its mechanism of action. Altogether, our findings support the concept that GM6 replicates the activity of a neurotrophic factor targeting developmental-stage pathways mediating neurogenesis. In these respects, GM6 appears to provide a small molecule with properties that have long been pursued in drug development for treatment of neurodegenerative disease [19, 103–105]. We have highlighted ways in which effects of GM6 may bolster neuron survival in the setting of ALS, although it is interesting to note that such effects may have therapeutic value for other diseases characterized by loss of neuronal cell populations (e.g., Alzheimer’s disease, Parkinson’s disease and Huntington disease). Neurotrophic factors such as glial cell line-derived neurotrophic factor (GDNF), brain-derived neurotrophic factor (BDNF), and neurturin, for example, all appear to protect against the striatonigral degeneration in Huntington’s disease [106–111]. The proposed neurotrophic effects of GM6 thus represents a general mechanism that, in the current era of single-target drug development [112], may provide a unique multi-target drug candidate for treatment of ALS and multiple other neurodegenerative conditions [103–105].

## Conclusions

ALS is a devastating disease with only three approved treatments available in the United States (riluzole, edaravone, and dextromethorphan/quinidine sulfate) [113]. No approved treatment significantly extends survival for ALS patients. GM604 has good drug-like properties [10, 17] and has demonstrated safety with promising effects in a small phase IIA clinical study [18]. This study used RNA-seq to provide the first complete analysis of gene expression responses to GM6 using the SH-SY5Y neuroblastoma model. Our findings demonstrate that GM6 alters the expression of 2867 protein-coding genes, which were frequently associated with developmental pathways linked to neurogenesis. We observed significant up-regulation of ligands, receptors and transcription factors associated with the Notch (*NOTCH1*, *NOTCH3*, *JAG2*, *HES7*) and hedgehog signaling pathways (*GLI1*, *GLI2*, *DHH*, *WNT6*). GM6 additionally altered the expression of genes associated with the extracellular matrix, mitochondria, inflammatory responses, mRNA processing and chromatin organization. We further characterized a network of DNA motifs and associated transcription factors potentially mediating transcriptional responses to GM6. The 2867 genes altered by GM6 includes 77 robustly associated with ALS by multiple sources, which were functionally important for neurogenesis, axon guidance and intrinsic apoptosis. These findings provide insights into mechanisms of action and support the hypothesis that GM6 acts upon developmental signaling pathways to promote neurotrophic effects and neuron survival. The regulation of multiple pathways and > 2800 genes by GM6 suggests a multi-target mechanism of action, which may ultimately be needed to treat and match the pathological complexity of ALS and other neurodegenerative conditions [20].

## Additional files

Additional file 1: Read mapping quality control (QC) assessment. (A) Number of reads per sample after QC filtering steps. (B) Percentage of reads mapped to the UCSC GRCh38/hg38 genome sequence. (C) Percentage of reads mapped to annotated genes. (D) Percentage of reads mapped to annotated exons. (E) Percentage of reads mapping to ribosomal sequences. (F) Number of protein-coding genes with detectable expression per sample. A gene was considered to have detectable expression if at least 1 read mapped to the gene’s sequence and if the FPKM 95% confidence interval lower limit was greater than 0. (TIF 1716 kb)

Additional file 2: Expression of protein-coding genes on each chromosome. (A) Average FPKM among genes located on each chromosome. (B) Average percentage of samples with detectable expression among genes located on each chromosome. A gene was considered to have detectable expression in a given sample if at least 1 read mapped to its sequence and if the FPKM 95% confidence interval lower bound was greater than 0. In (A) and (B), the number of protein-coding genes associated with each chromosome is listed in parentheses (bottom margin). (TIF 690 kb)

Additional file 3: Cluster and principal component analyses. (A) Hierarchical cluster analysis. The 28 samples were clustered based upon the expression of 14,569 protein-coding genes with detectable expression in at least 10 of the 28 samples (33%). Cluster analysis was performed using average linkage and the Euclidean distance metric. (B) PC plot. The 28 samples are plotted with respect to the first two principal component axes. The outlying sample “CTL-48 h-1” is indicated in the lower right corner. (C) PC plot (without outlier). The outlying sample “CTL-48 h-1” was removed and the 27 remaining samples are plotted with respect to the first 2 principal component axes. (TIF 1372 kb)

Additional file 4: Differential expression analyses. (A, D, G, J) Volcano plots. Log10-transformed *p*-values (vertical axis) are compared to FC estimates at each time point (horizontal axis). (B, E, H, K) MA plots. FC estimates (vertical axis) are compared to the average abundance of each gene (CPM = count per million, horizontal axis). (C, F, I, L) FPKM scatterplots. The average log10-transformed FPKM estimate was compared between GM6 and CTL treatments for all protein-coding gene with detectable expression. In (A) – (L), each point represents an individual gene. The number of DEGs identified in each analysis is indicated in the upper margin (FDR < 0.10 with FC > 1.50 or FC < 1.50). (TIF 1342 kb)

Additional file 5: RT-PCR validation of RNA-seq findings. (A, B) Calcium voltage-gated channel subunit alpha1 G (*CACNA1G*). (C, D) RIPOR family member 3 (*FAM65C*/*RIPOR3*). (E, F) Transmembrane protein 255A (*TMEM255A*). Panels (A), (C) and (E) show results from RNA-seq analyses (FPKM). Letters shown for each bar indicate results from post hoc treatment comparisons (Fisher’s least significant difference), where treatments not sharing the same letter differ significantly (*P* < 0.05). Panels (B), (D) and (F) show results from RT-PCR analyses (48 h time point). Average relative gene expression is shown using heat shock protein 90 alpha family class B member 1 (*HSP90AB1*) as a reference gene. The vertical axis for relative gene expression is arbitrary but normalized such that average expression of the CTL treatment is equal to 1. In (B) and (D), a one-tailed two-sample t-test was used to compare gene expression between the GM6 and CTL treatment (*P < 0.05; p-value is listed in the bottom margin). For panel (F), a one-tailed Wilcoxon rank sum test was used to compare gene expression between GM6 and CTL treatments (*P < 0.05; p-value is listed in the bottom margin; *P* = 0.064 based upon a one-tailed two-sample t-test). (TIF 517 kb)

Additional file 6: Gene Ontology (GO) cell component (CC), Kyoto Encyclopedia of Genes and Genomes (KEGG), Reactome and Disease Ontology (DO) terms associated with GM6-increased genes. (A, E, I, M) Top-ranked GO CC terms. Figures list GO CC terms most strongly enriched with respect to the GM6-increased DEGs identified at (A) 6 h, (E) 24 h, (I) 48 h and (M) 6–48 h. (B, F, J, N) Top ranked KEGG terms. Figures list KEGG terms most strongly enriched with respect to the GM6-increased DEGs identified at (B) 6 h, (F) 24 h, (J) 48 h and (N) 6–48 h. (C, G, K, O) Top-ranked Reactome terms. Figures list Reactome terms most strongly enriched with respect to the GM6-increased DEGs identified at (C) 6 h, (G) 24 h, (K) 48 h and (O) 6–48 h. (D, H, L, P) Top-ranked DO terms. Figures list DO terms most strongly enriched with respect to the GM6-increased DEGs identified at (D) 6 h, (H) 24 h, (L) 48 h and (P) 6–48 h. In (A) – (P), the analyzed DEGs were significant at the threshold of FDR < 0.10 and FC > 1.50. The number of GM6-increased genes associated with each term is listed in parentheses (left margin) and exemplar genes for each term are listed in each figure. Statistical significance of enrichment (horizontal axis) was evaluated using a hypergeometric test. Labels associated with some terms are abbreviated. (TIF 3144 kb)

Additional file 7: KEGG Notch signaling pathway (hsa04330). Pathway components are color-coded to indicate associations with GM6-increased (red) or GM6-decreased (blue) genes. The color scale (bottom right) reflects signed log100-transformed p-values, with positive values indicating GM6-increased genes (red) and negative values indicated GM6-decreased genes (blue). (TIF 881 kb)

Additional file 8: KEGG Hedgehog signaling pathway (hsa04340). Pathway components are color-coded to indicate associations with GM6-increased (red) or GM6-decreased (blue) genes. The color scale (bottom right) reflects signed log100-transformed p-values, with positive values indicating GM6-increased genes (red) and negative values indicated GM6-decreased genes (blue). (TIF 1990 kb)

Additional file 9: Gene Ontology (GO) cell component (CC), Kyoto Encyclopedia of Genes and Genomes (KEGG), Reactome and Disease Ontology (DO) terms associated with GM6-decreased genes. (A, E, I, M) Top-ranked GO CC terms. Figures list GO CC terms most strongly enriched with respect to the GM6-decreased DEGs identified at (A) 6 h, (E) 24 h, (I) 48 h and (M) 6–48 h. (B, F, J, N) Top ranked KEGG terms. Figures list KEGG terms most strongly enriched with respect to the GM6-decreased DEGs identified at (B) 6 h, (F) 24 h, (J) 48 h and (N) 6–48 h. (C, G, K, O) Top-ranked Reactome terms. Figures list Reactome terms most strongly enriched with respect to the GM6-decreased DEGs identified at (C) 6 h, (G) 24 h, (K) 48 h and (O) 6–48 h. (D, H, L, P) Top-ranked DO terms. Figures list DO terms most strongly enriched with respect to the GM6-decreased DEGs identified at (D) 6 h, (H) 24 h, (L) 48 h and (P) 6–48 h. In (A) – (D), the analyzed DEGs were significant at the threshold of FDR < 0.10 and FC > 1.50. In (E) – (P), the analyzed DEGs were significant at the less stringent threshold of FDR < 0.10 and FC < 1.00. The number of GM6-decreased genes associated with each term is listed in parentheses (left margin) and exemplar genes for each term are listed in each figure. Statistical significance of enrichment (horizontal axis) was evaluated using a hypergeometric test. Labels associated with some terms are abbreviated. (TIF 3066 kb)

Additional file 10: DNA binding sites associated with GM6-regulated genes. Genes differentially expressed at each time point were analyzed to identify DNA binding sites enriched in 5000 BP upstream regions. GM6-increased (▲) and GM6-decreased (▼) genes were evaluated separately. The table lists the number of enriched DNA motifs identified (FDR < 0.05) from among 2935 screened (column 3). The protein interacting with the most significant binding site is shown (column 4) with its DNA binding site consensus sequence (column 5). Footnotes (a) – (h) list GM6-regulated genes (FDR < 0.10) known to interact with one or more of the significant motifs identified (ordered from most to least strongly altered by GM6). Footnotes (i) – (p) list GM6-regulated target genes with the greatest density of binding sites in the upstream region. (PDF 185 kb)

Additional file 11: DNA motifs enriched in sequences upstream of GM6-increased genes. (A – D) Figures show motifs enriched in 5000 bp regions upstream of genes increased by GM6 at (A) 6 h, (B) 24 h, (C) 48 h and (D) 6–48 h (FDR < 0.10). Motif labels and consensus sequences are listed in the left margin. Red font is used for motifs known to interact with a protein encoded by a GM6-increased gene (*P* < 0.05), and blue font used for motifs known to interact with a protein encoded by a GM6-decreased gene (P < 0.05). Sequence logos for the top-ranked 12 motifs are shown for each time point. (TIF 2072 kb)

Additional file 12: DNA motifs enriched in sequences upstream of GM6-decreased genes. (A – D) Figures show motifs enriched in 5000 bp regions upstream of genes decreased by GM6 at (A) 6 h, (B) 24 h, (C) 48 h and (D) 6–48 h (FDR < 0.10). Motif labels and consensus sequences are listed in the left margin. Red font is used for motifs known to interact with a protein encoded by a GM6-increased gene (P < 0.05), and blue font used for motifs known to interact with a protein encoded by a GM6-decreased gene (P < 0.05). Sequence logos for the top-ranked 12 motifs are shown for each time point. (TIF 2023 kb)

Additional file 13: DNA motifs enriched in sequences upstream of GM6-regulated genes and known to interact with a protein encoded by a GM6-regulated gene (6–48 h). (A) Motifs associated with GM6-increased genes. (B) Motifs associated with GM6-decreased genes. In (A) and (B), red font is used for motifs known to interact with a protein encoded by a GM6-increased gene (FDR < 0.10) and blue font is used for motifs known to interact with a protein encoded by a GM6-decreased gene (FDR < 0.10). Sequence logos for the top-ranked 12 motifs are shown for each analysis. (C) Signal transducer and activator of transcription 3 (*STAT3*) expression. (D) Zinc finger matrin-type 2 (*ZMAT2*) expression. (E) Annexin A11 (*ANXA11*) expression. (F) MYCN proto-oncogene bHLH transcription factor (*MYCN*) expression. (G) EEF1A lysine methyltransferase 3 (*METTL21B*) expression. (H) Homeobox D11 (*HOXD11*) expression. (I) Thyroid hormone receptor interactor 10 (*TRIP10*) expression. (J) CUGBP Elav-like family member 5 (*CELF5*) expression. In (C) – (J), letters shown for each bar indicate results from post hoc treatment comparisons (Fisher’s least significant difference), where treatments not sharing the same letter differ significantly (P < 0.05). (TIF 1637 kb)

Additional file 14: Gene databases used to identify ALS-associated genes. The table lists the 9 databases used to identify ALS-associated genes, the associated PubMed identifier, and the number of ALS-associated genes identified from each source. The bottom rows list the number of ALS-genes common to multiple database sources. (PDF 14 kb)

Additional file 15: Functional analysis of ALS-associated genes (2+ sources) with expression significantly altered by GM6 treatment (FDR < 0.10). (A, B) GO BP terms associated with (A) GM6-increased and (B) GM6-decreased genes. (C, D) GO CC terms associated with (C) GM6-increased and (D) GM6-decreased genes. (E, F) KEGG terms associated with (E) GM6-increased and (F) GM6-decreased genes. (G, H) KEGG terms associated with (G) GM6-increased and (H) GM6-decreased genes. For (A) – (H), GM6-increased genes include those significantly increased by GM6 with respect to any of the 4 differential expression analyses performed (FDR < 0.10; 6, 24, 48 and/or 6–48 h), while GM6-decreased genes include those significantly decreased by GM6 with respect to any of the 4 differential expression analyses performed (FDR < 0.10; 6, 24, 48 and/or 6–48 h). The number of ALS-associated and GM6-increased/decreased genes associated with each term is listed in parentheses (left margin) and exemplar genes for each term are listed in each figure. Statistical significance of enrichment (horizontal axis) was evaluated using a hypergeometric test. Labels associated with some terms are abbreviated. (TIF 2066 kb)

## Abbreviations

ALS: Amyotrophic lateral sclerosis
BP: Biological process
CC: Cell component
CTL: Control treatment
DEG: Differentially expressed gene
ECM: Extracellular matrix
FC: Fold-change
FDR: False discovery rate
FPKM: Fragments per kilobase of exon per million reads mapped
GAM: Generalized additive logistic model
GM6: GM604 hexapeptide (H-Phe-Ser-Arg-Tyr-Ala-Arg-OH)
GO: Gene ontology
KEGG: Kyoto Encyclopedia of Genes and Genomes
MOA: Mechanism of action
PC: Principal component
PWM: Position weight matrix
QC: Quality control
SOM: Self-organizing map
TF: Transcription factor
