# Correction: GM604 regulates developmental neurogenesis pathways and the expression of genes associated with amyotrophic lateral sclerosis

**DOI:** 10.1186/s40035-020-00207-0

**Published:** 2020-06-12

**Authors:** William R. Swindell, Krzysztof Bojanowski, Mark S. Kindy, Raymond M. W. Chau, Dorothy Ko

**Affiliations:** 1 Heritage College of Osteopathic Medicine, Ohio University, Athens, OH USA; 2 Sunny BioDiscovery, Inc, Santa Paula, CA USA; 3 Department of Pharmaceutical Sciences, College of Pharmacy, University of South Florida, Tampa, FL USA; 4 James A. Haley VAMC, Tampa, FL USA; 5 Genervon Biopharmaceuticals LLC, Pasadena, CA USA

**Correction to: Translational Neurodegeneration (2018) 7:30**

**https://doi.org/10.1186/s40035-018-0135-7**

The original version of this article [1] refers to a protein previously described from rat muscle and originally designated motoneuronotrophic factor 1 (MNTF1) [2]. Nucleotide and amino acid sequences for MNTF1 were submitted to GenBank on December 17, 2001 in association with US patent 6309877 (GeneBank IDs: AR175906.1, AR175907.1, AR175908.1, AAE85614.1, AAE85615.1) [3]. Between 2004 and 2009, additional nucleotide and protein sequences for MNTF1 were submitted to GenBank in association with subsequent patent applications (i.e, AR562526.1, AR562527.1, AR562528.1, AAV23796.1, AR630645.1, AR630646.1, AR630647.1, AAX01604.1, ABP12727.1, ABP12728.1, ABP12729.1, ABP12730.1, ABP12731.1, ABP12732.1, ABP12733.1, DJ051830.1, DJ051831.1, DJ051832.1, DJ051833.1, DJ051834.1, DJ051835.1, DJ051836.1, DJ051837.1, DJ051838.1, DJ051839.1, DJ051840.1, DJ051841.1, GP281736.1, GP281737.1, GP281738.1, ACQ12655.1, ACQ12656.1) [4–13]. All sequences were derived during patent prosecution by Genervon Biopharmaceuticals, LLC. Patent applications were submitted by co-author RMWC, by co-author DK, or in one instance [7] under contract service by a non-coauthor investigator.

The GM604 (Alirinetide) synthetic linear peptide sequence (H-Phe-Ser-Arg-Tyr-Ala-Arg-OH) evaluated in our study [1] was determined from these sequences and experiments as described in the above-cited patent applications [3–13]. Aside from these patents, however, there is limited evidence supporting GM604 as a true homologue of MNTF1 or the existence of MNTF1 in nature [14–22]. The synthetic linear peptide sequence GM604 therefore cannot be claimed as a true homologue of a naturally occurring protein. Further in vivo characterization of MNTF1 or MNTF1-related proteins thus remains an avenue for future work, although this was not an objective in our study [1], which focused on transcriptional responses to the synthetic hexapeptide defined above. This correction serves to provide relevant background information but does not affect results or conclusions from our reported study [1].
