# Supplementary figures and images for: GM604 regulates developmental neurogenesis pathways and the expression of genes associated with amyotrophic lateral sclerosis
Source: Transl Neurodegener. 2018 Dec 3;7:30. doi: 10.1186/s40035-018-0135-7 (PMC6276193; doi:10.1186/s40035-018-0135-7)

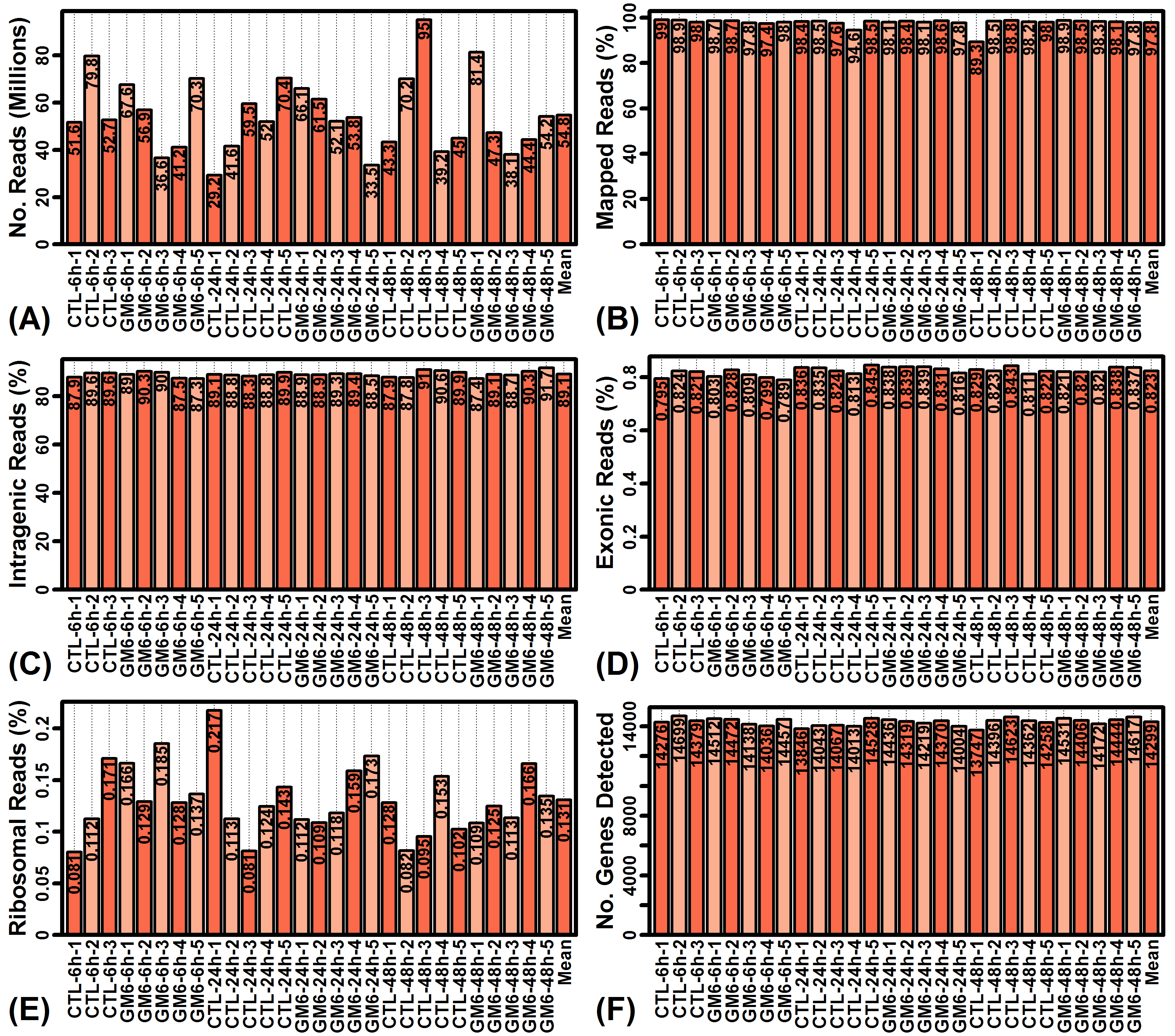

Supplement: Supplementary file 1 — Read mapping quality control (QC) assessment. (A) Number of reads per sample after QC filtering steps. (B) Percentage of reads mapped to the UCSC GRCh38/hg38 genome sequence. (C) Percentage of reads mapped to annotated genes. (D) Percentage of reads mapped to annotated exons. (E) Percentage of reads mapping to ribosomal sequences. (F) Number of protein-coding genes with detectable expression per sample. A gene was considered to have detectable expression if at least 1 read mapped to the gene’s sequence and if the FPKM 95% confidence interval lower limit was greater than 0. (TIF 1716 kb) [file 40035_2018_135_MOESM1_ESM.tif]

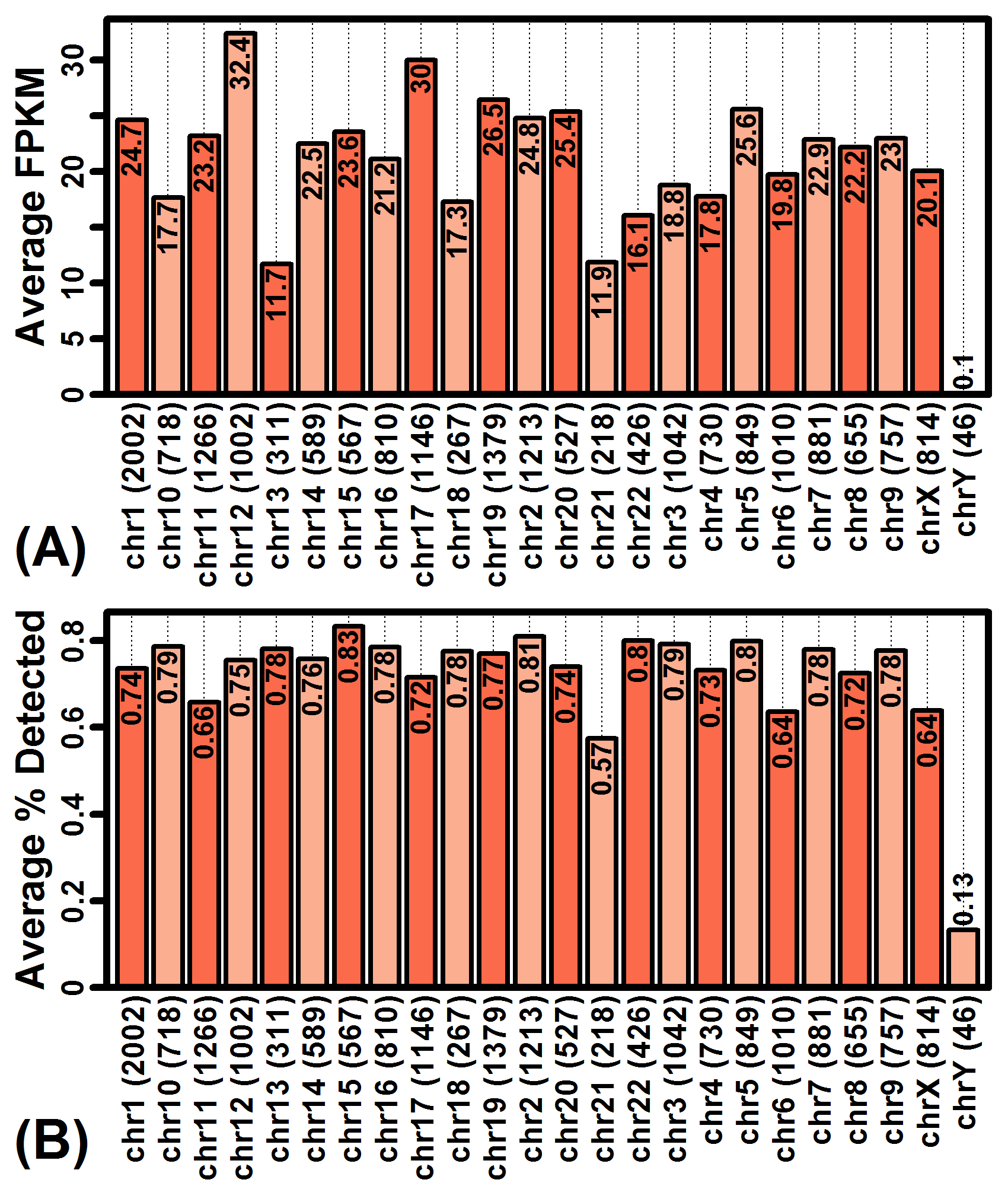

Supplement: Supplementary file 2 — Expression of protein-coding genes on each chromosome. (A) Average FPKM among genes located on each chromosome. (B) Average percentage of samples with detectable expression among genes located on each chromosome. A gene was considered to have detectable expression in a given sample if at least 1 read mapped to its sequence and if the FPKM 95% confidence interval lower bound was greater than 0. In (A) and (B), the number of protein-coding genes associated with each chromosome is listed in parentheses (bottom margin). (TIF 690 kb) [file 40035_2018_135_MOESM2_ESM.tif]

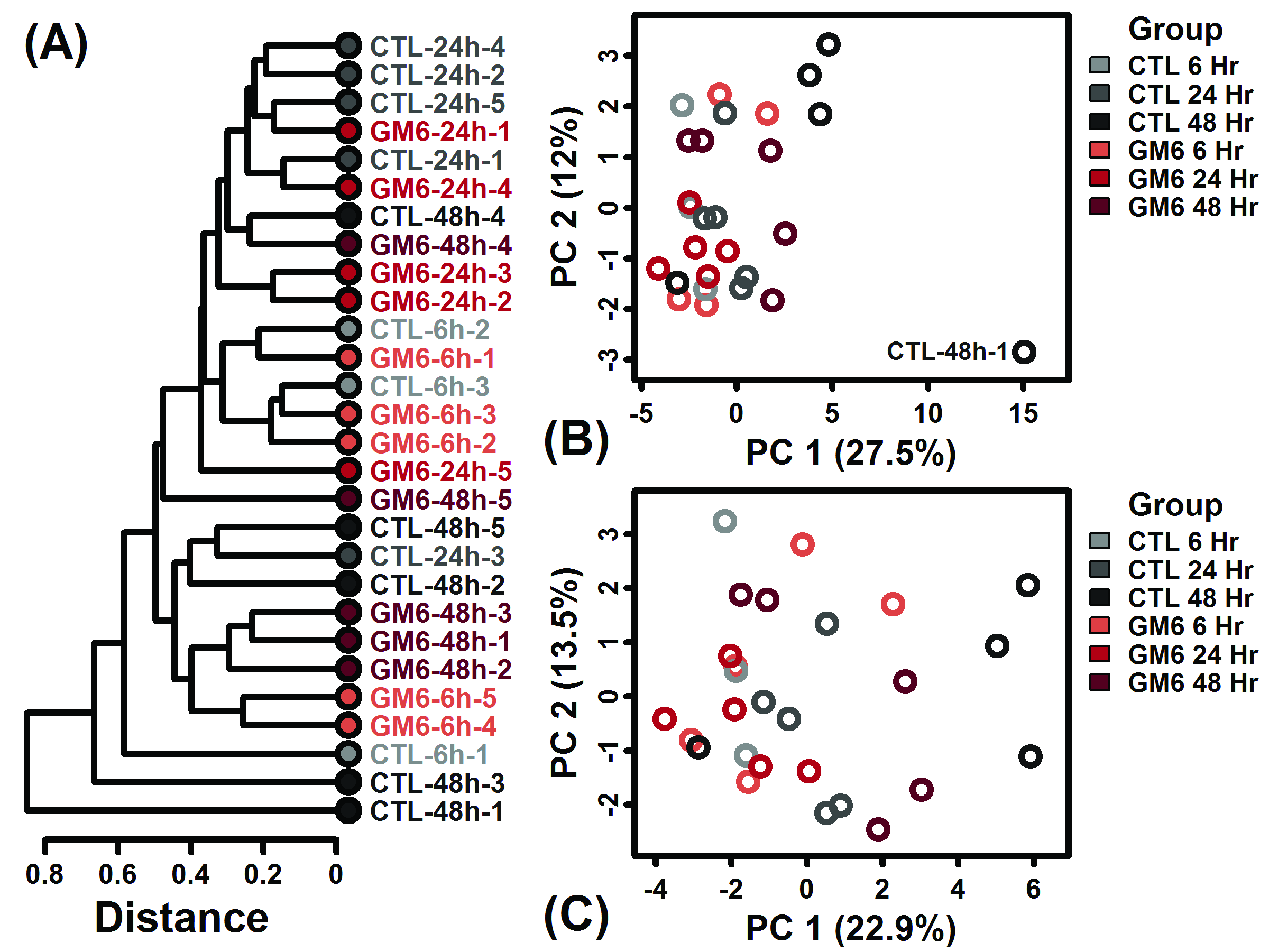

Supplement: Supplementary file 3 — Cluster and principal component analyses. (A) Hierarchical cluster analysis. The 28 samples were clustered based upon the expression of 14,569 protein-coding genes with detectable expression in at least 10 of the 28 samples (33%). Cluster analysis was performed using average linkage and the Euclidean distance metric. (B) PC plot. The 28 samples are plotted with respect to the first two principal component axes. The outlying sample “CTL-48 h-1” is indicated in the lower right corner. (C) PC plot (without outlier). The outlying sample “CTL-48 h-1” was removed and the 27 remaining samples are plotted with respect to the first 2 principal component axes. (TIF 1372 kb) [file 40035_2018_135_MOESM3_ESM.tif]

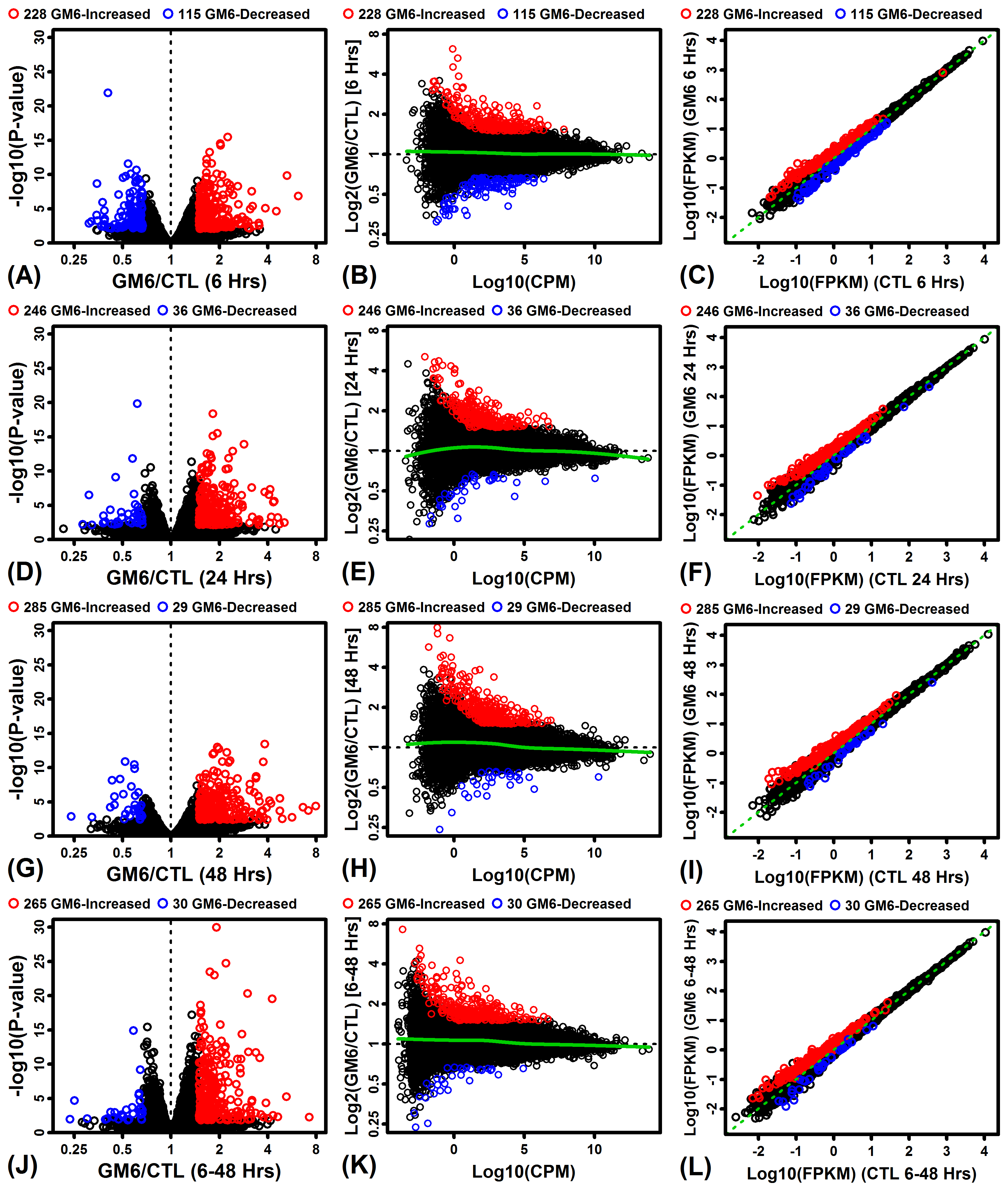

Supplement: Supplementary file 4 — Differential expression analyses. (A, D, G, J) Volcano plots. Log10-transformed p-values (vertical axis) are compared to FC estimates at each time point (horizontal axis). (B, E, H, K) MA plots. FC estimates (vertical axis) are compared to the average abundance of each gene (CPM = count per million, horizontal axis). (C, F, I, L) FPKM scatterplots. The average log10-transformed FPKM estimate was compared between GM6 and CTL treatments for all protein-coding gene with detectable expression. In (A) – (L), each point represents an individual gene. The number of DEGs identified in each analysis is indicated in the upper margin (FDR < 0.10 with FC > 1.50 or FC < 1.50). (TIF 1342 kb) [file 40035_2018_135_MOESM4_ESM.tif]

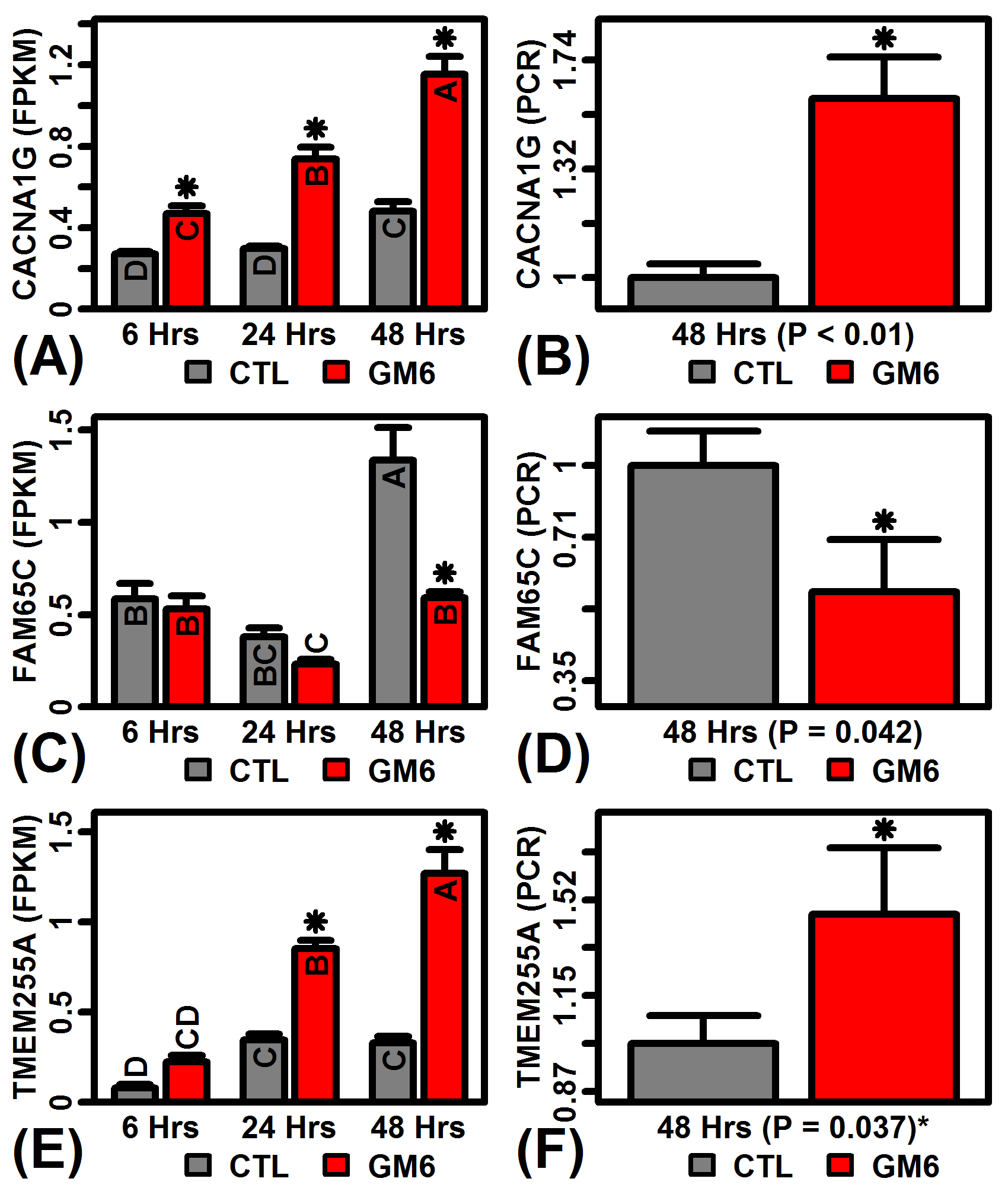

Supplement: Supplementary file 5 — RT-PCR validation of RNA-seq findings. (A, B) Calcium voltage-gated channel subunit alpha1 G (CACNA1G). (C, D) RIPOR family member 3 (FAM65C/RIPOR3). (E, F) Transmembrane protein 255A (TMEM255A). Panels (A), (C) and (E) show results from RNA-seq analyses (FPKM). Letters shown for each bar indicate results from post hoc treatment comparisons (Fisher’s least significant difference), where treatments not sharing the same letter differ significantly (P < 0.05). Panels (B), (D) and (F) show results from RT-PCR analyses (48 h time point). Average relative gene expression is shown using heat shock protein 90 alpha family class B member 1 (HSP90AB1) as a reference gene. The vertical axis for relative gene expression is arbitrary but normalized such that average expression of the CTL treatment is equal to 1. In (B) and (D), a one-tailed two-sample t-test was used to compare gene expression between the GM6 and CTL treatment (*P < 0.05; p-value is listed in the bottom margin). For panel (F), a one-tailed Wilcoxon rank sum test was used to compare gene expression between GM6 and CTL treatments (*P < 0.05; p-value is listed in the bottom margin; P = 0.064 based upon a one-tailed two-sample t-test). (TIF 517 kb) [file 40035_2018_135_MOESM5_ESM.tif]

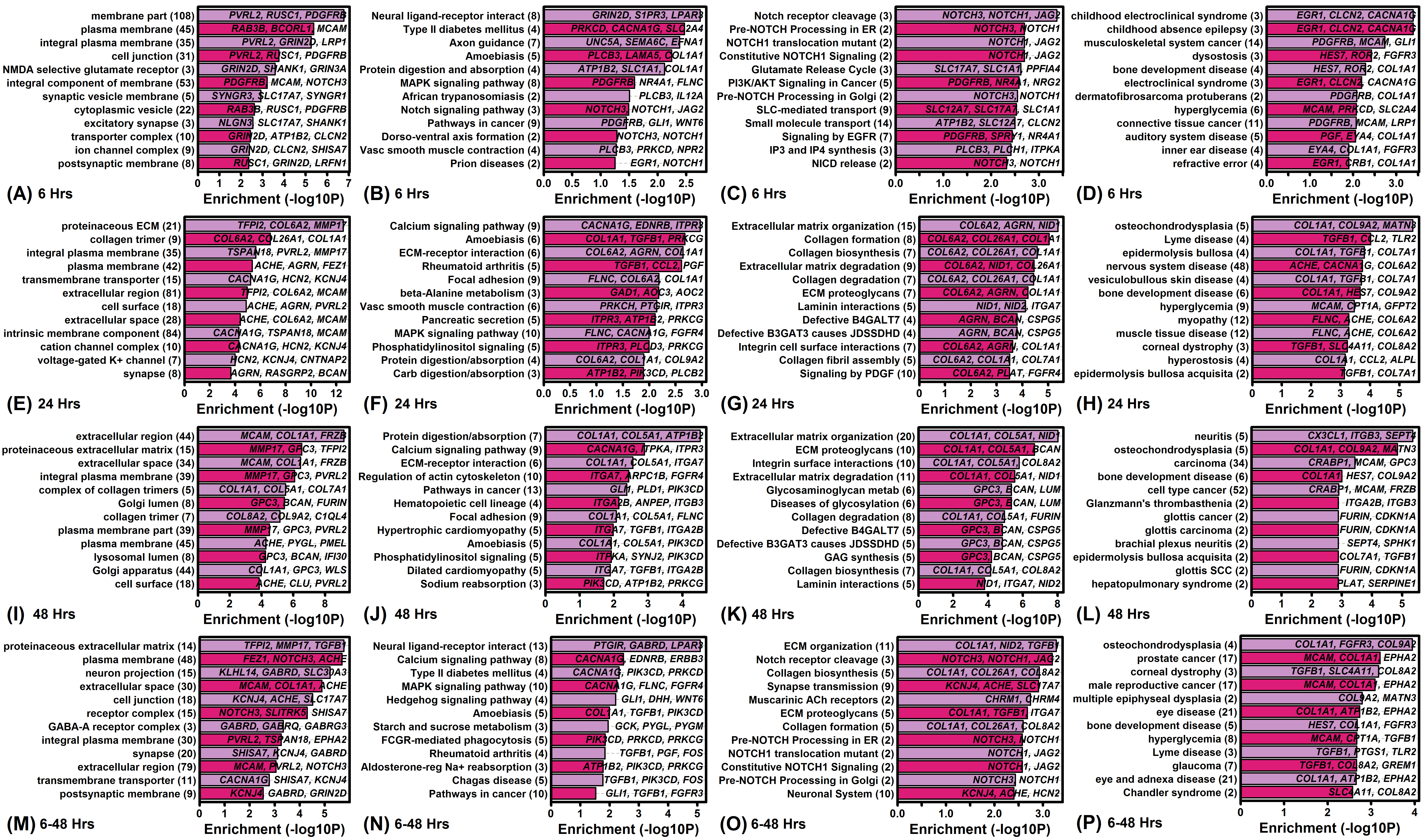

Supplement: Supplementary file 6 — Gene Ontology (GO) cell component (CC), Kyoto Encyclopedia of Genes and Genomes (KEGG), Reactome and Disease Ontology (DO) terms associated with GM6-increased genes. (A, E, I, M) Top-ranked GO CC terms. Figures list GO CC terms most strongly enriched with respect to the GM6-increased DEGs identified at (A) 6 h, (E) 24 h, (I) 48 h and (M) 6–48 h. (B, F, J, N) Top ranked KEGG terms. Figures list KEGG terms most strongly enriched with respect to the GM6-increased DEGs identified at (B) 6 h, (F) 24 h, (J) 48 h and (N) 6–48 h. (C, G, K, O) Top-ranked Reactome terms. Figures list Reactome terms most strongly enriched with respect to the GM6-increased DEGs identified at (C) 6 h, (G) 24 h, (K) 48 h and (O) 6–48 h. (D, H, L, P) Top-ranked DO terms. Figures list DO terms most strongly enriched with respect to the GM6-increased DEGs identified at (D) 6 h, (H) 24 h, (L) 48 h and (P) 6–48 h. In (A) – (P), the analyzed DEGs were significant at the threshold of FDR < 0.10 and FC > 1.50. The number of GM6-increased genes associated with each term is listed in parentheses (left margin) and exemplar genes for each term are listed in each figure. Statistical significance of enrichment (horizontal axis) was evaluated using a hypergeometric test. Labels associated with some terms are abbreviated. (TIF 3144 kb) [file 40035_2018_135_MOESM6_ESM.tif]

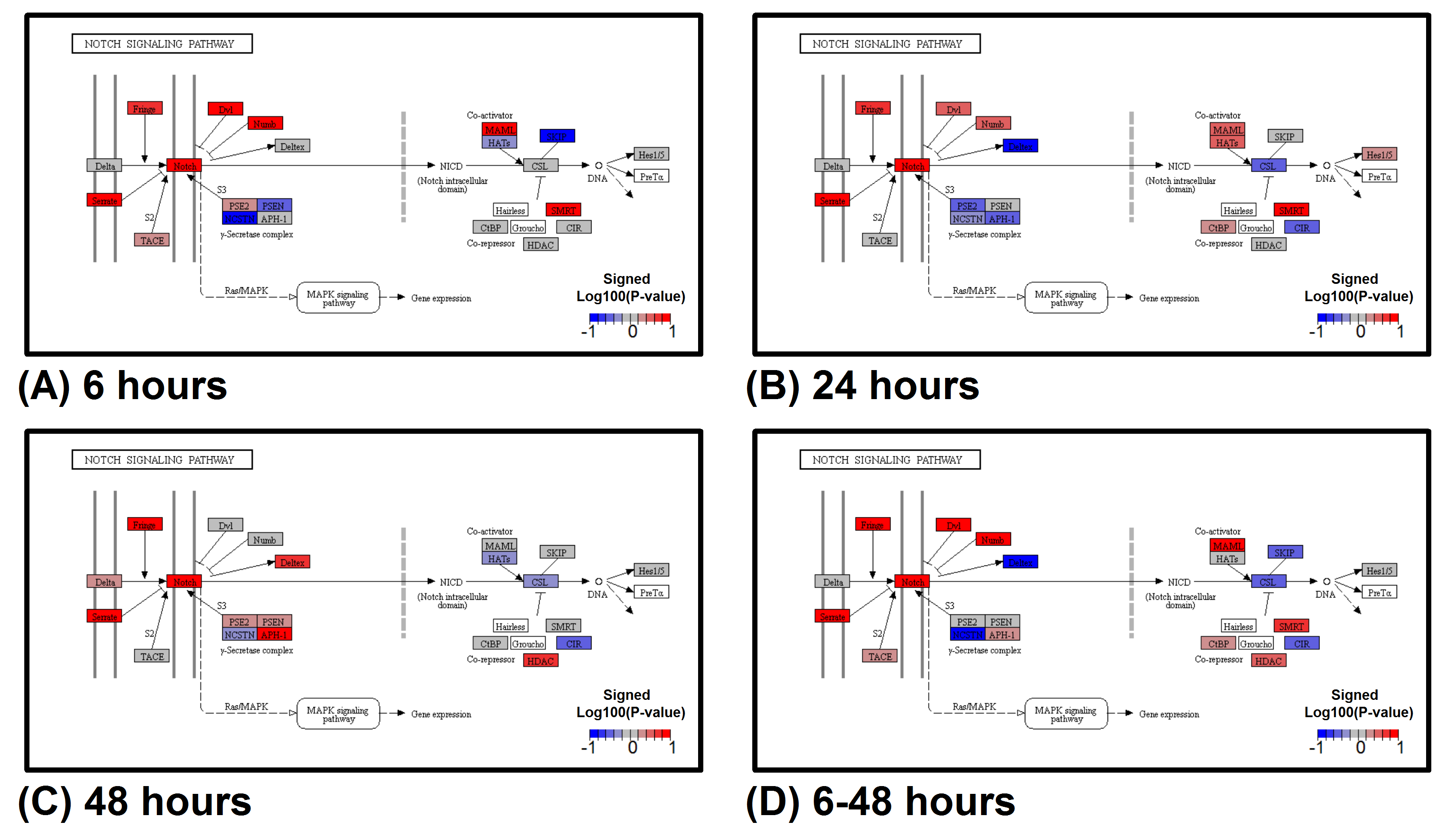

Supplement: Supplementary file 7 — KEGG Notch signaling pathway (hsa04330). Pathway components are color-coded to indicate associations with GM6-increased (red) or GM6-decreased (blue) genes. The color scale (bottom right) reflects signed log100-transformed p-values, with positive values indicating GM6-increased genes (red) and negative values indicated GM6-decreased genes (blue). (TIF 881 kb) [file 40035_2018_135_MOESM7_ESM.tif]

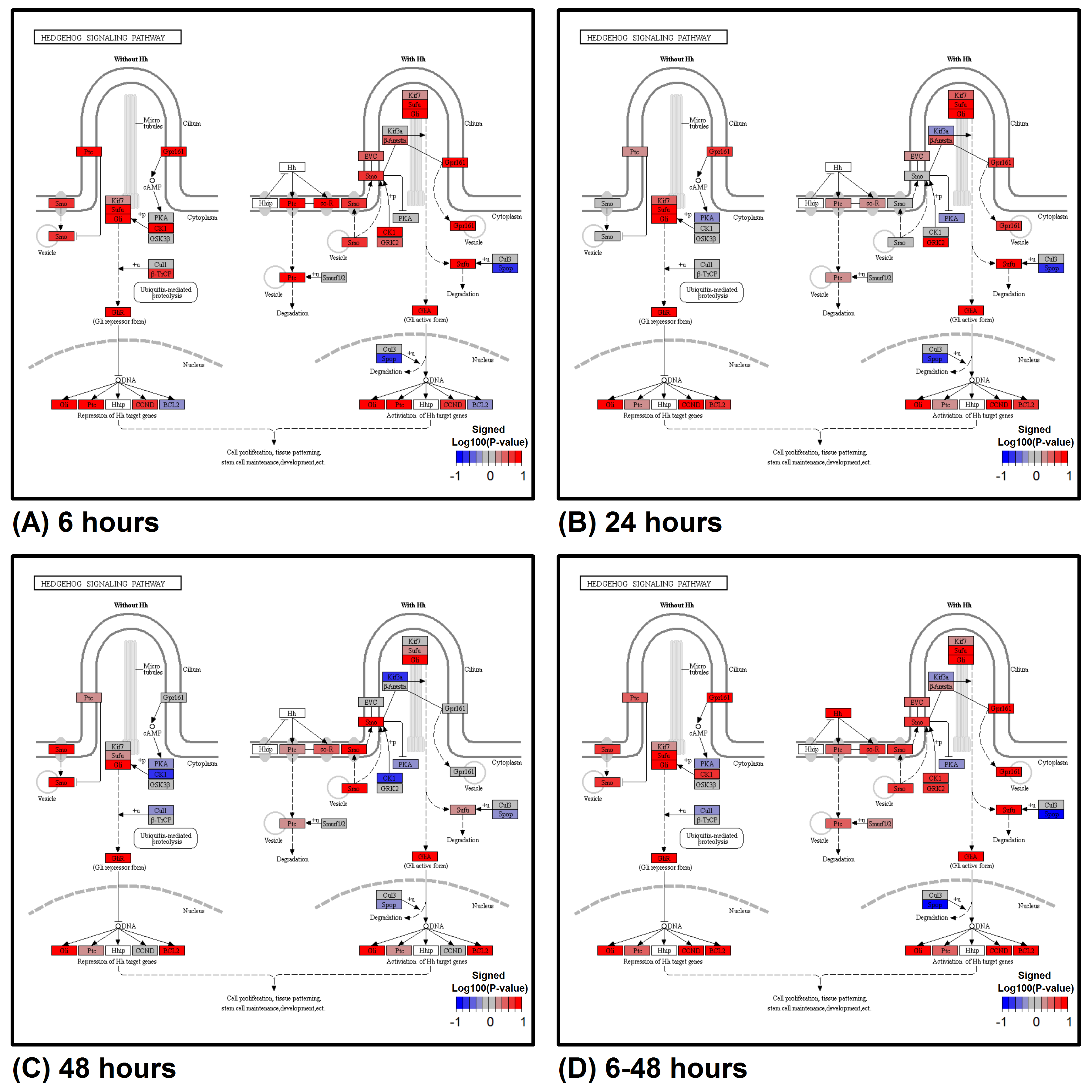

Supplement: Supplementary file 8 — KEGG Hedgehog signaling pathway (hsa04340). Pathway components are color-coded to indicate associations with GM6-increased (red) or GM6-decreased (blue) genes. The color scale (bottom right) reflects signed log100-transformed p-values, with positive values indicating GM6-increased genes (red) and negative values indicated GM6-decreased genes (blue). (TIF 1990 kb) [file 40035_2018_135_MOESM8_ESM.tif]

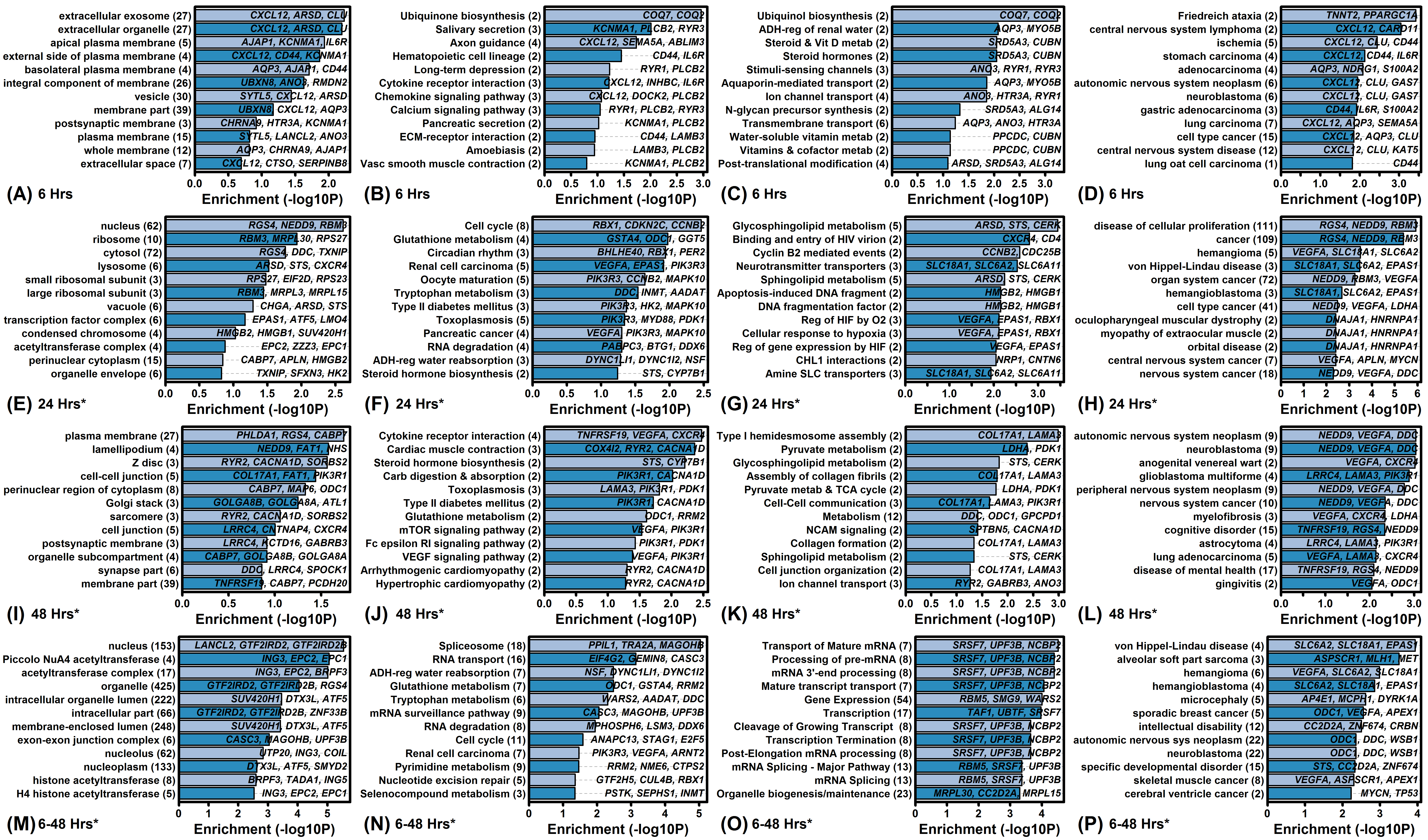

Supplement: Supplementary file 9 — Gene Ontology (GO) cell component (CC), Kyoto Encyclopedia of Genes and Genomes (KEGG), Reactome and Disease Ontology (DO) terms associated with GM6-decreased genes. (A, E, I, M) Top-ranked GO CC terms. Figures list GO CC terms most strongly enriched with respect to the GM6-decreased DEGs identified at (A) 6 h, (E) 24 h, (I) 48 h and (M) 6–48 h. (B, F, J, N) Top ranked KEGG terms. Figures list KEGG terms most strongly enriched with respect to the GM6-decreased DEGs identified at (B) 6 h, (F) 24 h, (J) 48 h and (N) 6–48 h. (C, G, K, O) Top-ranked Reactome terms. Figures list Reactome terms most strongly enriched with respect to the GM6-decreased DEGs identified at (C) 6 h, (G) 24 h, (K) 48 h and (O) 6–48 h. (D, H, L, P) Top-ranked DO terms. Figures list DO terms most strongly enriched with respect to the GM6-decreased DEGs identified at (D) 6 h, (H) 24 h, (L) 48 h and (P) 6–48 h. In (A) – (D), the analyzed DEGs were significant at the threshold of FDR < 0.10 and FC > 1.50. In (E) – (P), the analyzed DEGs were significant at the less stringent threshold of FDR < 0.10 and FC < 1.00. The number of GM6-decreased genes associated with each term is listed in parentheses (left margin) and exemplar genes for each term are listed in each figure. Statistical significance of enrichment (horizontal axis) was evaluated using a hypergeometric test. Labels associated with some terms are abbreviated. (TIF 3066 kb) [file 40035_2018_135_MOESM9_ESM.tif]

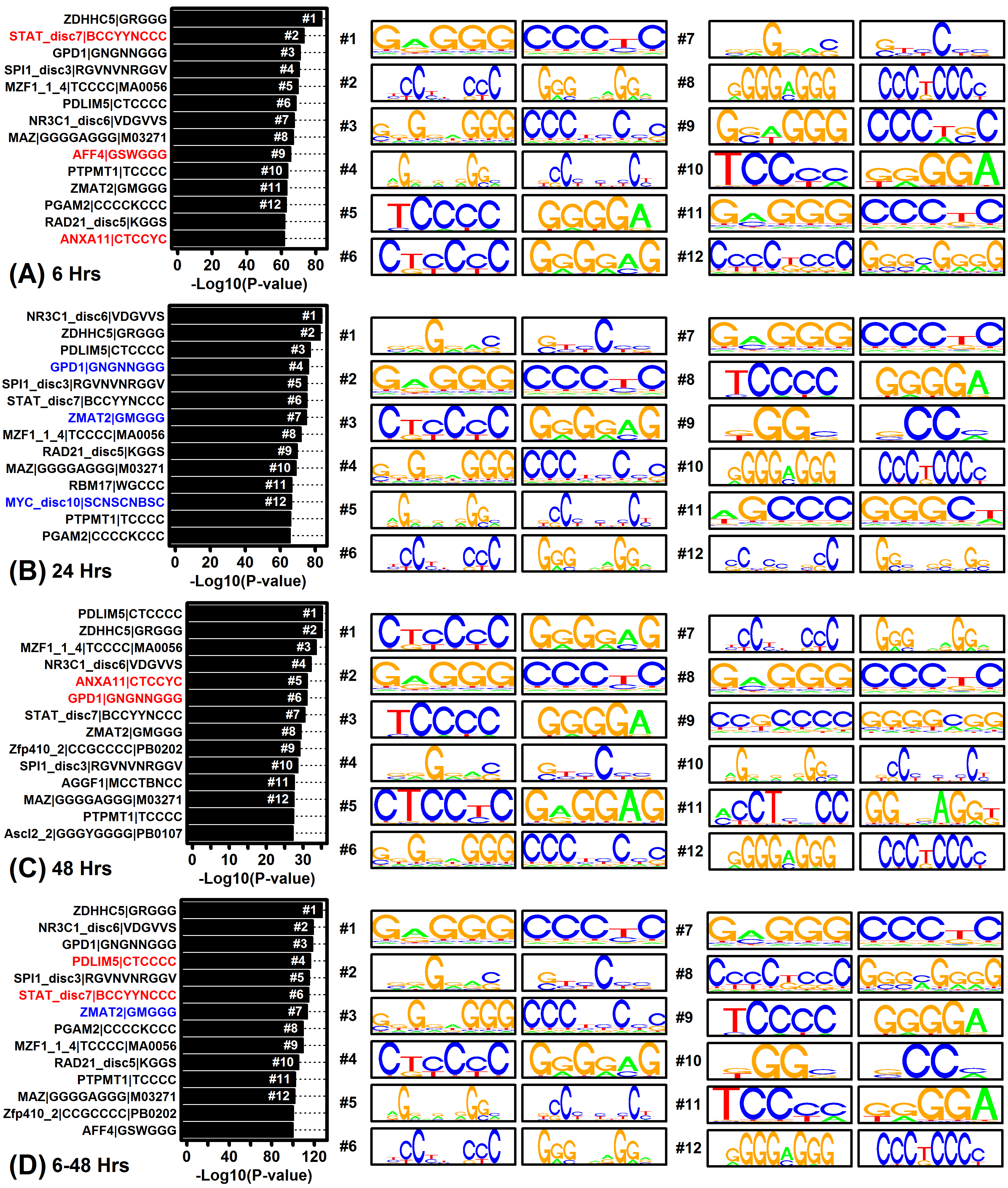

Supplement: Supplementary file 11 — DNA motifs enriched in sequences upstream of GM6-increased genes. (A – D) Figures show motifs enriched in 5000 bp regions upstream of genes increased by GM6 at (A) 6 h, (B) 24 h, (C) 48 h and (D) 6–48 h (FDR < 0.10). Motif labels and consensus sequences are listed in the left margin. Red font is used for motifs known to interact with a protein encoded by a GM6-increased gene (P < 0.05), and blue font used for motifs known to interact with a protein encoded by a GM6-decreased gene (P < 0.05). Sequence logos for the top-ranked 12 motifs are shown for each time point. (TIF 2072 kb) [file 40035_2018_135_MOESM11_ESM.tif]

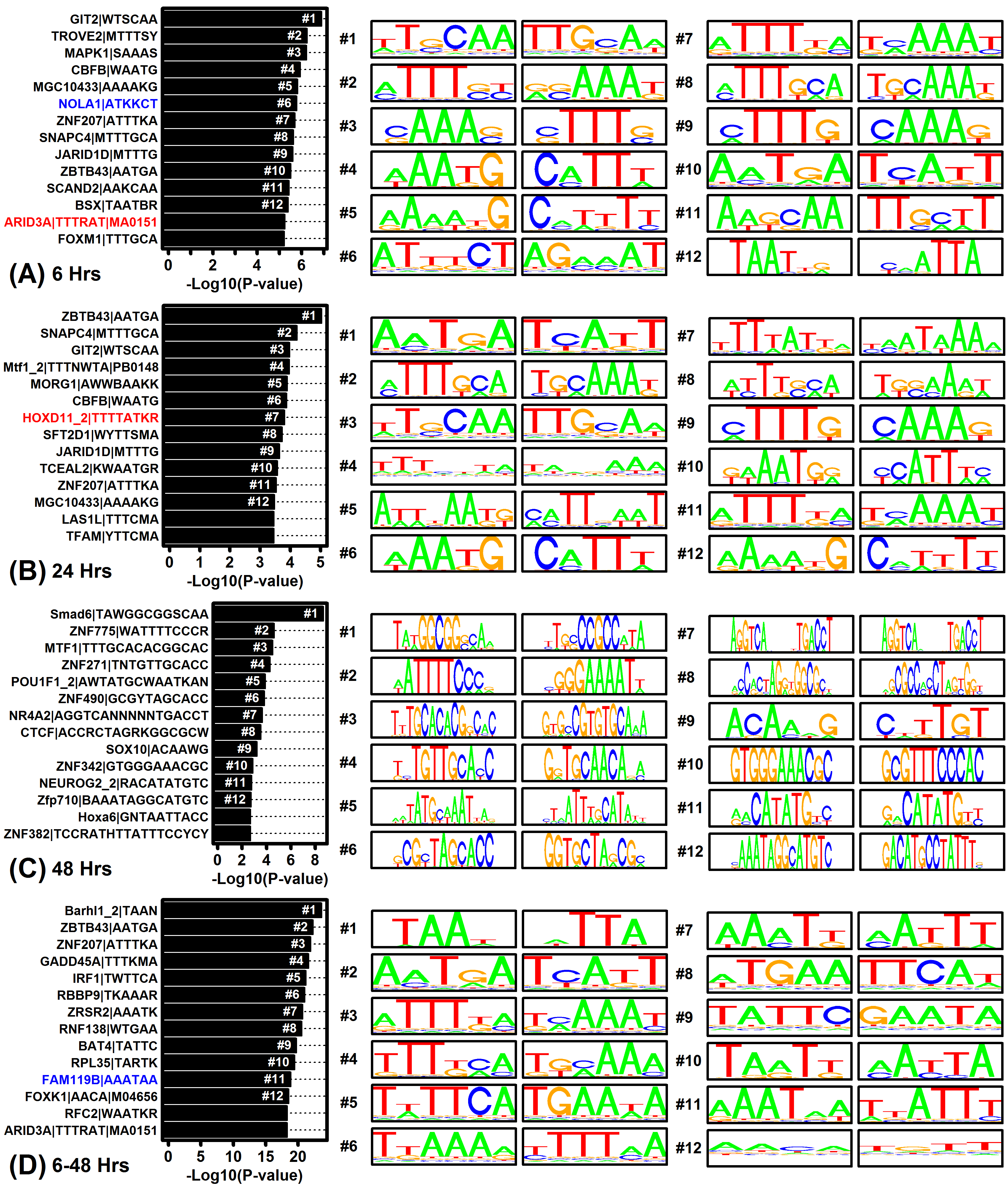

Supplement: Supplementary file 12 — DNA motifs enriched in sequences upstream of GM6-decreased genes. (A – D) Figures show motifs enriched in 5000 bp regions upstream of genes decreased by GM6 at (A) 6 h, (B) 24 h, (C) 48 h and (D) 6–48 h (FDR < 0.10). Motif labels and consensus sequences are listed in the left margin. Red font is used for motifs known to interact with a protein encoded by a GM6-increased gene (P < 0.05), and blue font used for motifs known to interact with a protein encoded by a GM6-decreased gene (P < 0.05). Sequence logos for the top-ranked 12 motifs are shown for each time point. (TIF 2023 kb) [file 40035_2018_135_MOESM12_ESM.tif]

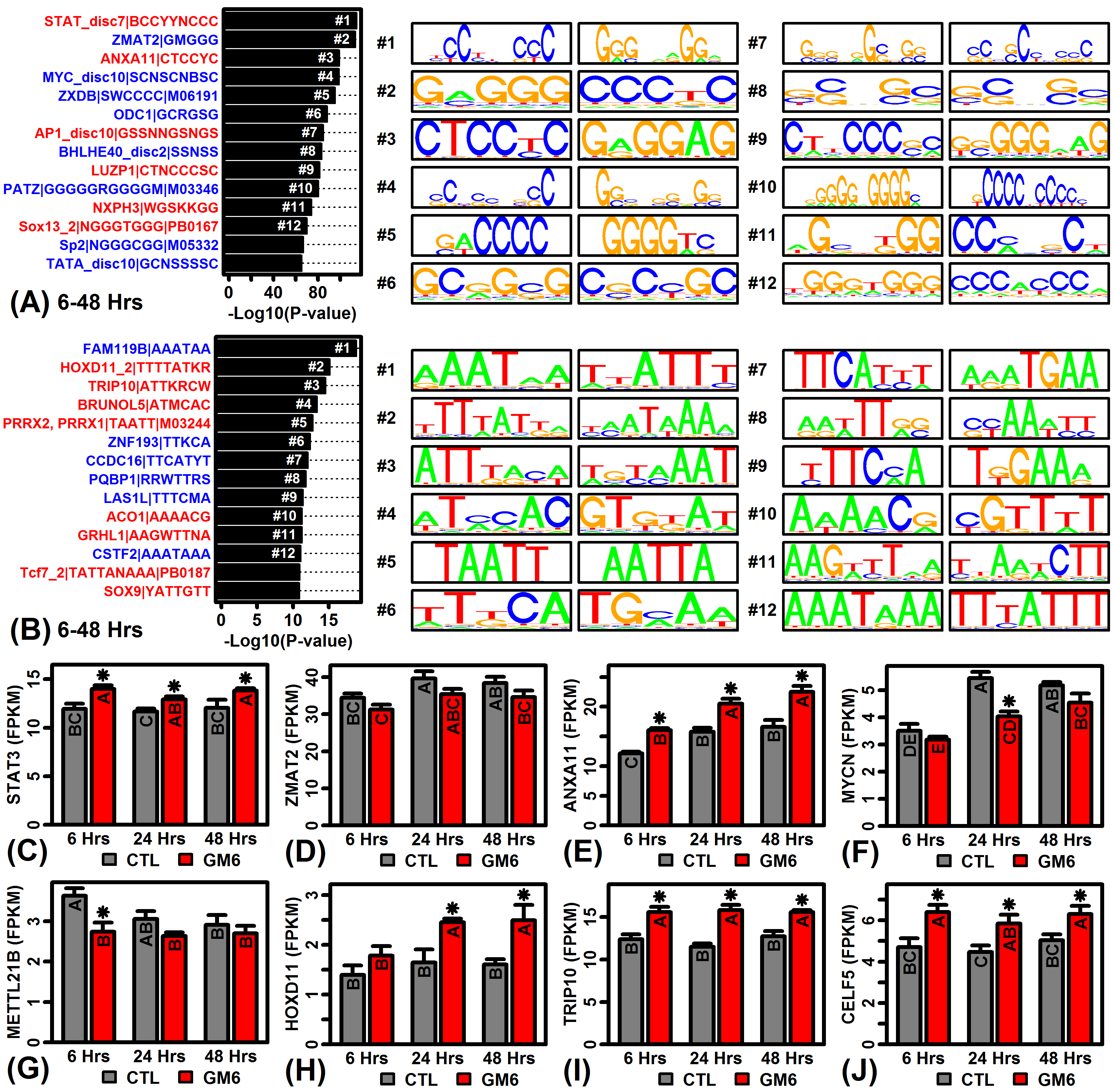

Supplement: Supplementary file 13 — DNA motifs enriched in sequences upstream of GM6-regulated genes and known to interact with a protein encoded by a GM6-regulated gene (6–48 h). (A) Motifs associated with GM6-increased genes. (B) Motifs associated with GM6-decreased genes. In (A) and (B), red font is used for motifs known to interact with a protein encoded by a GM6-increased gene (FDR < 0.10) and blue font is used for motifs known to interact with a protein encoded by a GM6-decreased gene (FDR < 0.10). Sequence logos for the top-ranked 12 motifs are shown for each analysis. (C) Signal transducer and activator of transcription 3 (STAT3) expression. (D) Zinc finger matrin-type 2 (ZMAT2) expression. (E) Annexin A11 (ANXA11) expression. (F) MYCN proto-oncogene bHLH transcription factor (MYCN) expression. (G) EEF1A lysine methyltransferase 3 (METTL21B) expression. (H) Homeobox D11 (HOXD11) expression. (I) Thyroid hormone receptor interactor 10 (TRIP10) expression. (J) CUGBP Elav-like family member 5 (CELF5) expression. In (C) – (J), letters shown for each bar indicate results from post hoc treatment comparisons (Fisher’s least significant difference), where treatments not sharing the same letter differ significantly (P < 0.05). (TIF 1637 kb) [file 40035_2018_135_MOESM13_ESM.tif]

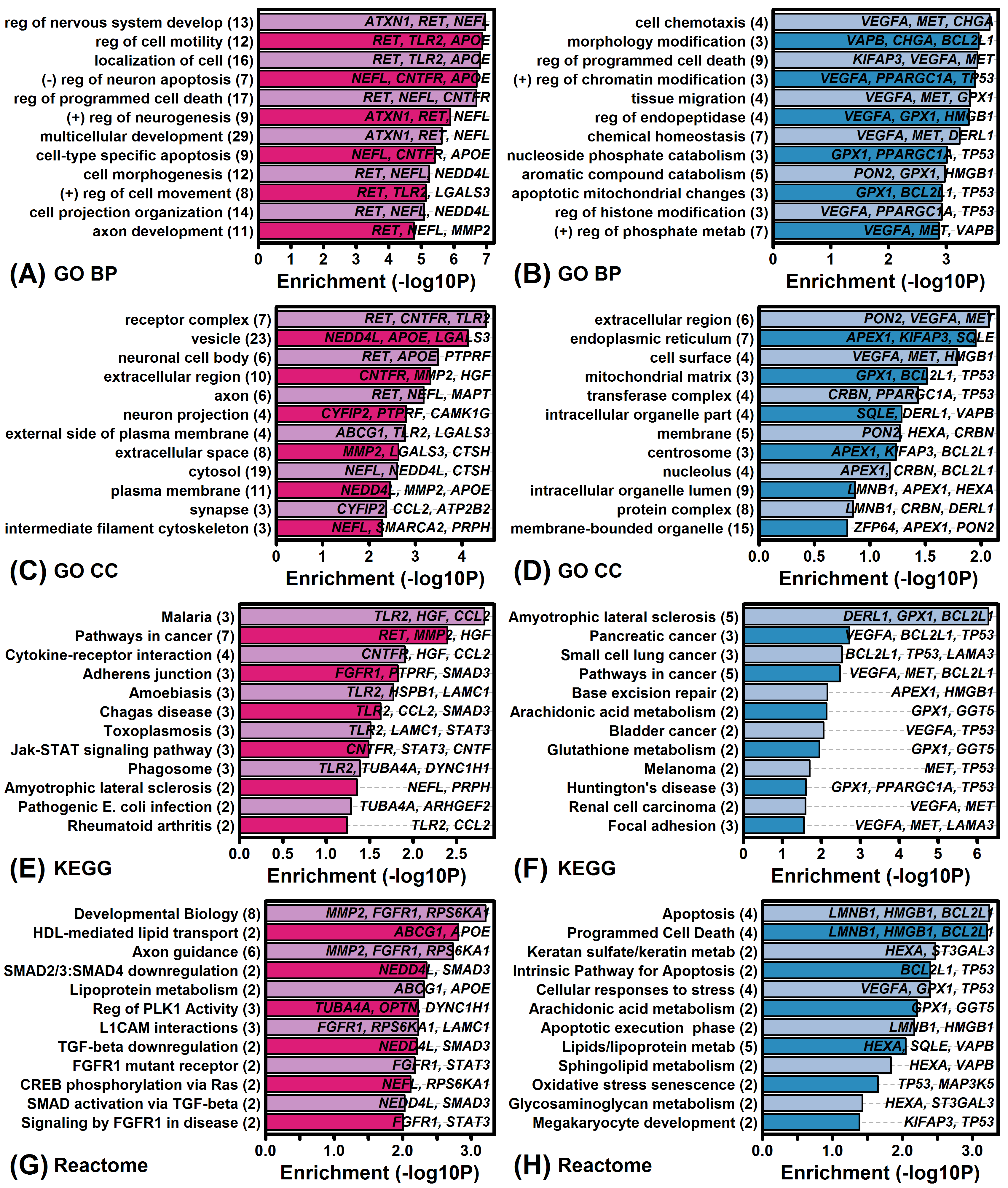

Supplement: Supplementary file 15 — Functional analysis of ALS-associated genes (2+ sources) with expression significantly altered by GM6 treatment (FDR < 0.10). (A, B) GO BP terms associated with (A) GM6-increased and (B) GM6-decreased genes. (C, D) GO CC terms associated with (C) GM6-increased and (D) GM6-decreased genes. (E, F) KEGG terms associated with (E) GM6-increased and (F) GM6-decreased genes. (G, H) KEGG terms associated with (G) GM6-increased and (H) GM6-decreased genes. For (A) – (H), GM6-increased genes include those significantly increased by GM6 with respect to any of the 4 differential expression analyses performed (FDR < 0.10; 6, 24, 48 and/or 6–48 h), while GM6-decreased genes include those significantly decreased by GM6 with respect to any of the 4 differential expression analyses performed (FDR < 0.10; 6, 24, 48 and/or 6–48 h). The number of ALS-associated and GM6-increased/decreased genes associated with each term is listed in parentheses (left margin) and exemplar genes for each term are listed in each figure. Statistical significance of enrichment (horizontal axis) was evaluated using a hypergeometric test. Labels associated with some terms are abbreviated. (TIF 2066 kb) [file 40035_2018_135_MOESM15_ESM.tif]
