# Supplementary material for: GM604 regulates developmental neurogenesis pathways and the expression of genes associated with amyotrophic lateral sclerosis
Source: Transl Neurodegener. 2018 Dec 3;7:30. doi: 10.1186/s40035-018-0135-7 (PMC6276193; doi:10.1186/s40035-018-0135-7)
Supplement: Supplementary file 10 — DNA binding sites associated with GM6-regulated genes. Genes differentially expressed at each time point were analyzed to identify DNA binding sites enriched in 5000 BP upstream regions. GM6-increased (▲) and GM6-decreased (▼) genes were evaluated separately. The table lists the number of enriched DNA motifs identified (FDR < 0.05) from among 2935 screened (column 3). The protein interacting with the most significant binding site is shown (column 4) with its DNA binding site consensus sequence (column 5). Footnotes (a) – (h) list GM6-regulated genes (FDR < 0.10) known to interact with one or more of the significant motifs identified (ordered from most to least strongly altered by GM6). Footnotes (i) – (p) list GM6-regulated target genes with the greatest density of binding sites in the upstream region. (PDF 185 kb) [file 40035_2018_135_MOESM10_ESM.pdf]

Additional File 10. DNA binding sites associated with GM6-regulated genes. Genes differentially expressed at each time point were analyzed to identify DNA binding sites enriched in 5000 BP upstream regions. GM6-increased (▲) and GM6-decreased (▼) genes were evaluated separately. The table lists the number of enriched DNA motifs identified (FDR < 0.05) from among 2935 screened (column 3). The protein interacting with the most significant binding site is shown (column 4) with its DNA binding site consensus sequence (column 5). Footnotes (a) – (h) list GM6-regulated genes (FDR < 0.10) known to interact with one or more of the significant motifs identified (ordered from most to least strongly altered by GM6). Footnotes (i) – (p) list GM6-regulated target genes with the greatest density of upstream binding sites.

Time	Direction	No. Motifs	Protein	DNA binding site
6 h	▲	550[a]	ZDHHC5	5-GAGGG/CCCTC-3[i]
	▼	229[b]	GIT2	5-TTGCAA/TTGCAA-3[j]
24 h	▲	591[c]	NR3C1	5-GGGGAC/GTCCCC-3[k]
	▼	23[d]	ZBTB43	5-AATGA/TCATT-3[l]
48 h	▲	448[e]	PDLIM5	5-CTCCCC/GGGGAG-3[m]
	▼	4[f]	SMAD6	5-TATGGCGGGCAA/TTGCCCGCCATA-3[n]
6 – 48 h	▲	656[g]	ZDHHC5	5-GAGGG/CCCTC-3[o]
	▼	498[h]	BARHL1	5-TAAT/ATTA-3[p]

[a]GM6-increased: EGR1, ANXA11, ZBTB7B, ZNF284, TFEB, LUZP1, THRA, FOS, IRF3, ATF3, SOX13, FOXP4, NFIX, BCL6B, MEIS3, ZNF423, TRIM69, ZFHX2, PAX5, AFF4; GM6-decreased: ODC1, SP2, ZFP64, SPAG7, HLCS, PATZ1, TFCP2, NCBP2, DUS3L, ZBTB14, C9orf156, MYC, KLF11, USF2, ZNF184, TAF1, ZNF304, ZNF341, FIZ1, ZNF443, PPARGC1A

[b]GM6-increased: AGAP2, HOXC6, IRF3, FEZ1, TRIP10, CELF5, NFIX, TCEAL2; GM6-decreased: ZNF33B, ZNF76, LAS1L, ZNF124

[c]GM6-increased: SOX13, ANXA11, JDP2, RREB1, ZBTB7B, FOXP4, EGR1, ID4, TAGLN2; GM6-decreased: MYCN, IKZF1, BHLHE40, ODC1, KLF11, ZNF160, ZNF789, FOSL2, ZNF256, ZXDB

[d]None of the 23 motifs interacted were known to interact with proteins encoded by genes differentially expressed with GM6 treatment (FDR < 0.10).

[e]GM6-increased: SOX13, MYLK, ANXA11, RREB1; GM6-decreased: KLF11, ODC1, IKZF1, FOSL2, TAF1, BHLHE40, ZNF286B

[f]None of the 4 motifs interacted were known to interact with proteins encoded by genes differentially expressed with GM6 treatment (FDR < 0.10).

[g]GM6-increased: SOX13, ANXA11, ZBTB7B, FOXP4, RREB1, NXPH3, THRA, JDP2, IRF5, TFEB, SIX4, MEIS3, STAT3, SREBF1, EZR, CD59, FOS, SMAD3, FOSL1, EGR1, LUZP1, ZNF423, ZNF467, IRF3, ETS1, GLI2, BCL3, CERS4, ATF3, ID4; GM6-decreased: ODC1, KLF11, IKZF1, TAF1, ZNF304, ZNF160, ZNF266, MYCN, BHLHE40, ZFP64, ZNF532, DIABLO, ZBTB40, CBX3, NCBP2, SPAG7, ZXDB, FAM127B, ZMAT2, ZSCAN31, MYC, ZNF184, ZFX, ZNF443, ZNF383, ESRRG, FIZ1, PATZ1, ZNF595, NRL, SP2, VEZF1

[h]GM6-increased: FEZ1, ARID5B, SOX13, TRIP10, FOXO6, RAX, MAP4K2, CELF5, TPPP, ACO1, AGAP2, ZBTB46, ARID5A, TCF7, HOXD11, SOX9, STAT3, GRHL1, FOSL1, ELF3, SOX5, IRF3, GATA4, MYLK, CEBPA; GM6-decreased: RBM3, ZNF286B, ZSCAN9, ZNF124, ZFP30, SLC18A1, LAS1L, HNRNPA1, ASPSCR1, ZNF830, TRMT1, CLK1, TSN, ZNF184, PSMA6, METTL21B, CDK2AP1, ZNF75A, PQBP1, CSTF2, PICK1
[i]Targets: GRIN2D, CPLX1, ZBTB7B, NLGN2, SHANK1
[j]Targets: PCDHB10, GALNT12, NAP1L3, DNAH5, COQ2
[k]Targets: CPLX1, FBXL16, GRIN2D, C2CD4C, CBX4
[l]Targets: PCDHB9, EIF2A, PCDHA9, LAMP5, LRRN3
[m]Targets: GAS6, FGFRL1, LMNA, MCAM, RARG
[n]Targets: GTF2IRD2, DDC, GTF2IRD2B, GOLGA8B, GOLGA8A
[o]Targets: GRIN2D, IGLON5, CPLX1, ZBTB7B, TNNI3
[p]Targets: PCDHB10, PCDHB9, PCDHGA6, MPZL3, SLC9B2
