# Supplementary material for: GM604 regulates developmental neurogenesis pathways and the expression of genes associated with amyotrophic lateral sclerosis
Source: Transl Neurodegener. 2018 Dec 3;7:30. doi: 10.1186/s40035-018-0135-7 (PMC6276193; doi:10.1186/s40035-018-0135-7)
Supplement: Supplementary file 14 — Gene databases used to identify ALS-associated genes. The table lists the 9 databases used to identify ALS-associated genes, the associated PubMed identifier, and the number of ALS-associated genes identified from each source. The bottom rows list the number of ALS-genes common to multiple database sources. (PDF 14 kb) [file 40035_2018_135_MOESM14_ESM.pdf]

Additional File 14. Gene databases used to identify ALS-associated genes. The table lists the 9 databases used to identify ALS-associated genes, the associated PubMed identifier, and the number of ALS-associated genes identified from each source. The bottom rows list the number of ALS-genes common to multiple database sources.

**Source**	**Pubmed Reference**	**No. ALS-associated Genes**
1. NHGRI-EBI GWAS Catalog[a]	27899670	349
2. MeSH Database[b]	25887539	873
3. Disease Ontology[c]	25348409	155
4. DisGeNET[d]	27924018	75
5. KEGG Database[e]	27899662	51
6. eDGAR Database[f]	28812536	27
7. MalaCards[g]	27899610	155
8. ALSOD[h]	18608099	126
9. ALSGene[i]	21702733	17
Common to 2+ sources	---	383
Common to 3+ sources	---	133
Common to 4+ sources	---	59
Common to 5+ sources	---	36
Common to 6+ sources	---	21
Common to 7+ sources	---	4
Common to 8+ sources[j]	---	1
Common to 9 sources	---	0

[a]Database of genome-wide association studies (GWAS) reported since 2008 (www.ebi.ac.uk/gwas/).
[b]Database of Medical Subject Headings (MeSH) terms based upon annotations of PubMed documents. ALS-associated genes were identified based upon the MeSH term D000544 (https://www.ncbi.nlm.nih.gov/mesh).
[c]Disease Ontology is a disease-centered database with genes organized according to disease etiology (http://www.disease-ontology.org). ALS-associated genes were identified based upon the DO identifier 10652.
[d]DisGeNET provides a comprehensive catalogue of genes and variants associated to human diseases (http://www.disgenet.org).
[e]Kyoto Encyclopedia of Genes and Genomes (KEGG). ALS-associated genes were identified based upon the KEGG pathway identifier hsa05010 (http://www.kegg.jp/).
[f]Database of Disease-Gene Associations (eDGAR) (edgar.biocomp.unibo.it). The eDGAR database integrates gene-disease associations based upon the OMIM, HUMSAVAR and CLINVAR databases.
[g]Integrated compendium of annotated diseases mined from 68 data sources (http://www.malacards.org/).
[h]Amyotrophic Lateral Sclerosis Online Genetics Database (ALSOD). (alsod.iop.kcl.ac.uk/)
[i]ALS Gene Database. A comprehensive collection of published genetic association studies assessing ALS risk. (http://www.alsgene.org/)
[j]Superoxide dismutase 1 (*SOD1*) was common to 8 of 9 database sources.
